# Adaptive Metabolic Responses Facilitate Blood‐Brain Barrier Repair in Ischemic Stroke via BHB‐Mediated Epigenetic Modification of ZO‐1 Expression

**DOI:** 10.1002/advs.202400426

**Published:** 2024-04-26

**Authors:** Ruijie Li, Yilin Liu, Jihao Wu, Xiong Chen, Qiying Lu, Kai Xia, Congyuan Liu, Xin Sui, Yixuan Liu, Yiling Wang, Yuan Qiu, Jinsi Chen, Yi Wang, Ruijun Li, Yucheng Ba, Jiayun Fang, Weijun Huang, Zhengqi Lu, Yanbing Li, Xinxue Liao, Andy Peng Xiang, Yinong Huang

**Affiliations:** ^1^ Center for Stem Cell Biology and Tissue Engineering Key Laboratory for Stem Cells and Tissue Engineering Ministry of Education Sun Yat‐Sen University Guangzhou 510080 China; ^2^ National‐Local Joint Engineering Research Center for Stem Cells and Regenerative Medicine Zhongshan School of Medicine Sun Yat‐sen University Guangzhou 510080 China; ^3^ Department of Endocrinology The First Affiliated Hospital of Sun Yat‐Sen University Guangzhou 510080 China; ^4^ Department of Urology Sun Yat‐sen Memorial Hospital Sun Yat‐Sen University Guangzhou 510120 China; ^5^ Department of Rehabilitation Medicine The Third Affiliated Hospital Sun Yat‐sen University 600 Tianhe Road Guangzhou 510630 China; ^6^ Department of Surgery Intensive Care Unit The Third Affiliated Hospital of Sun Yat‐Sen University Guangzhou 510630 China; ^7^ Guangdong Institute for Drug Control NMPA Key Laboratory for Quality Control of Blood Products Guangdong Drug Administration Key Laboratory of Quality Control and Research of Blood Products Guangzhou 510663 China; ^8^ Department of Neurology The Third Affiliated Hospital Sun Yat‐sen University 600 Tianhe Road Guangzhou 510630 China; ^9^ Department of Cardiology The First Affiliated Hospital of Sun Yat‐Sen University 58 Zhongshan 2nd Road Guangzhou 510080 China; ^10^ Department of Histoembryology and Cell Biology Zhongshan School of Medicine Sun Yat‐sen University Guangzhou 510080 China; ^11^ Guangdong Provincial Key Laboratory of Diabetology Guangzhou 510630 China

**Keywords:** adipose, blood brain barrier (BBB), ketone body, lysine β‐hydroxybutyrylation (Kbhb), ZO‐1

## Abstract

Adaptive metabolic responses and innate metabolites hold promising therapeutic potential for stroke, while targeted interventions require a thorough understanding of underlying mechanisms. Adiposity is a noted modifiable metabolic risk factor for stroke, and recent research suggests that it benefits neurological rehabilitation. During the early phase of experimental stroke, the lipidomic results showed that fat depots underwent pronounced lipolysis and released fatty acids (FAs) that feed into consequent hepatic FA oxidation and ketogenesis. Systemic supplementation with the predominant ketone beta‐hydroxybutyrate (BHB) is found to exert discernible effects on preserving blood‐brain barrier (BBB) integrity and facilitating neuroinflammation resolution. Meanwhile, blocking FAO‐ketogenesis processes by administration of CPT1α antagonist or shRNA targeting HMGCS2 exacerbated endothelial damage and aggravated stroke severity, whereas BHB supplementation blunted these injuries. Mechanistically, it is unveiled that BHB infusion is taken up by monocarboxylic acid transporter 1 (MCT1) specifically expressed in cerebral endothelium and upregulated the expression of tight junction protein ZO‐1 by enhancing local β‐hydroxybutyrylation of H3K9 at the promoter of TJP1 gene. Conclusively, an adaptive metabolic mechanism is elucidated by which acute lipolysis stimulates FAO‐ketogenesis processes to restore BBB integrity after stroke. Ketogenesis functions as an early metabolic responder to restrain stroke progression, providing novel prospectives for clinical translation.

## Graphical abstract

Adiposity is a well‐known metabolic risk factor for stroke, but its role in promoting post‐stroke functional recovery remains unexplored. Li et al. reveal that stroke instigates adaptive lipolysis and subsequent FAO‐ketogenesis processes, promoting early blood‐brain barrier preservation by epigenetically regulating the tight junction protein ZO‐1 expression. These findings highlight the critical role of adaptive metabolic responses in post‐stroke neurorehabilitation.

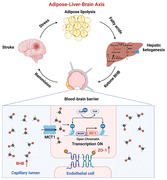

## 1 Introduction

Stroke ranks among the top three leading causes of global mortality and disability, representing a serious threat to public health.^[^ ^1^ ^]^ During the first few hours and days after stroke onset, the neurological deficit severity and clinical course of stroke patients are particularly unstable, which highly correlates with their long‐term functional recovery and clinical outcomes.^[^ ^2^ ^]^ Multiple risk factors and endogenous adaptive mechanisms are considered to contribute to this early neurological instability,^[^ ^3^ ^]^ and therapeutic approaches leveraging the power of early adaptive responses are receiving substantial interest.

Adiposity, a well‐documented modifiable risk factor for stroke occurrence, has been associated with conflicting findings regarding its impacts on stroke outcomes. Several large cohort studies have reported lower mortality rates in overweight or obesity stroke patients, when compared to their normal weight or underweight counterparts.^[^ ^4^ ^]^ Although the salutary impacts of adiposity on stroke outcomes remains incompletely understood, it is hypothesized that adaptive metabolic responses could partially explain this observation.^[^ ^5^ ^]^ Critical illness, such as acute cerebral injury, is characterized by systemic neuroendocrine activation including the overactive hypothalamus‐pituitary‐adrenal (HPA) axis and sympathetic nervous system (SNS).^[^ ^6^ ^]^ These elevated stress hormones invoke an overall catabolic/anabolic imbalance and exert potent lipolytic impacts on adipose tissue, which adaptively functions as an alternative energy source for vital organs.^[^ ^6^, ^7^ ^]^ Moreover, various pro‐inflammation lipid mediators generated by tissue injures contributes to the temporal dynamic shift of lipids metabolism, such as prostaglandins, leukotrienes and interleukins.^[^ ^8^ ^]^ Compared to lean individuals, diet‐induced obese (DIO) mice have been reported to exhibit enhanced lipid mobilization from adipose tissue and increased fatty acid oxidation (FAO) in metabolic tissues including liver and skeletal muscle.^[^ ^9^ ^]^ This increased lipid availability from excessive adipose is found to mediate protection against intensive care unit (ICU) acquired weakness during sepsis.^[^ ^10^ ^]^ In the model of acute neurological injury, mice exhibited significant reduction of adipose mass and distinct circulating lipid profile along with the development of  ischemic stroke, possibly attributable to the enzymatic activities of ATGL, HSL, MGL, and PLA2.^[^ ^11^ ^]^ Moreover, recent studies have identified several neuroprotective lipids metabolites, such as 15‐hydroxy‐eicosatrienoic acid and docosahexaenoic acid.^[^ ^11^, ^12^ ^]^ However, a thorough understanding of morphological, physiological, and metabolic alterations of adipose tissue following stroke is still lacking. Additionally, the potential pathophysiological implications and main executors of the systemic metabolic responses remain to be elaborated.

Ketone bodies (KBs), of which beta‐hydroxybutyrate (BHB) is the most abundant in circulation, are predominantly synthesized in the liver from fatty acid‐derived acetyl‐coenzyme A (acetyl‐CoA) mobilized from adipose tissue.^[^ ^13^ ^]^ This ketogenesis process has been recognized as an endogenous protective mechanism against energy crisis after critical illness by providing an alternative fuel to glucose for extra‐hepatic tissues such as muscle, kidney, heart, and brain.^[^ ^14^ ^]^ Since the brain is largely unable to directly utilize FAs, uptake of KBs from the extracellular space or circulation has been postulated to preserve higher brain function when glucose is scarce.^[^ ^15^ ^]^ BHB is known to be brain permeable and enter the brain dependent on its plasma levels.^[^ ^16^ ^]^ Monocarboxylate transporters (MCTs) are responsible for transportation of monocarboxylates such as lactates, pyruvates, and ketone body.^[^ ^17^ ^]^ Among these isoforms, MCT1 is found to play a pivotal role in ketolytic process, and its mutation is correlated with serious ketoacidosis.^[^ ^18^ ^]^ In addition to serving as highly efficient energetic substrates in response to acute stress, KBs have also been implicated as signaling mediators that coordinate cell fate decision and critical cellular events.^[^ ^19^ ^]^ In this regard, KBs exert multiple beneficial effects through uptake by MCTs or binding with G‐protein‐coupled receptors (GPRs) such as hydroxy‐carboxylic acid receptor 2 (HCA2, GPR109a) and free fatty acid receptor 3 (FFAR3, GPR41).^[^ ^20^ ^]^ In the cytoplasm, KBs have been unveiled to suppress the assembly and activation of NLR family pyrin domain‐containing 3 (NLRP3) inflammasome, reduce interleukin‐1β (IL‐1β) generation, and dampen pro‐inflammatory responses.^[^ ^21^ ^]^ Additionally, KBs could also enter the nucleus and induce epigenetic modifications on the transcriptional activity of target genes by inhibiting Class I endogenous histone deacetylases (HDACs) activity or enhancing histone β‐hydroxybutyrylation.^[^ ^22^ ^]^ Recent studies have unveiled the multifactorial roles of ketone in neuroprotection. As an alternative energy substrate, BHB enhances mitochondria respiratory, relieves oxidative stress, thereby ameliorating neuronal damage.^[^ ^23^ ^]^ Moreover, BHB has been found to induce a neuroprotective phenotype by inhibiting NF‐κB and NLRP3 signaling and facilitating resolution of neuroinflammation.^[^ ^24^ ^]^ Despite the considerable progress in ketone research, the pharmacological significance and molecular mechanism by which KBs orchestrate early rehabilitation after stroke remain to be elucidated.

In the current study, we delineate stroke‐induced massive adipose lipolysis and subsequent hepatic FAO‐ketogenesis and explore the beneficial effects of these adaptive metabolic responses in promoting early resolution of neuroinflammation. Mechanistically, we claim that lipid mobilization and KBs export exert robust neuroprotective effects in middle cerebral artery occlusion (MCAO) mice through epigenetic modification of the tight junction protein ZO‐1 at BBB.

## 2 Results

### 2.1 Ischemic Stroke Instigates Acute Lipolysis and Browning in White Adipose Tissue (WAT)

**Figure 1:**
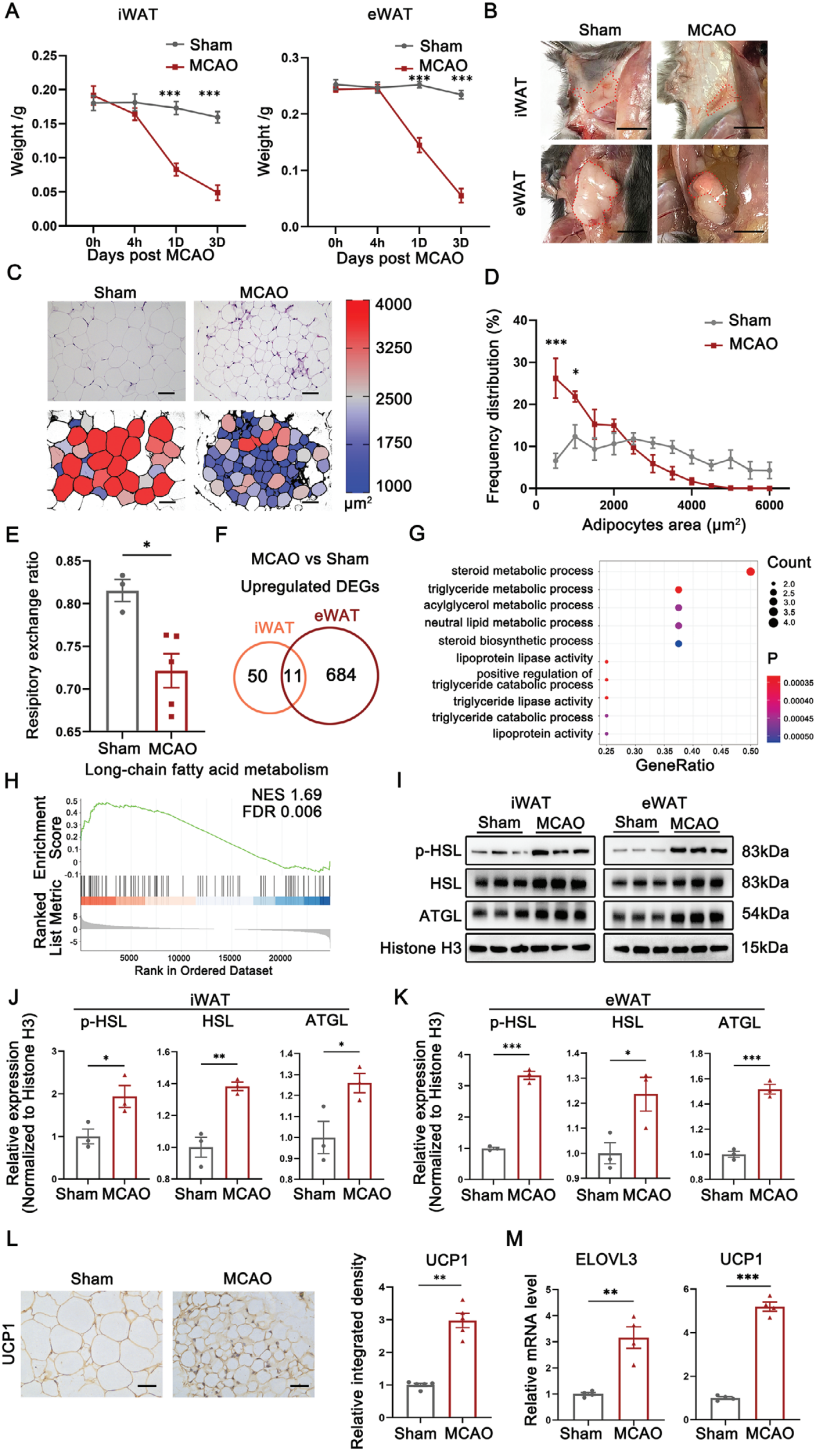
Ischemic stroke instigates acute lipolysis and browning in white adipose tissue (WAT). A) Temporal dynamics of iWAT and eWAT weight alterations after stroke. n = 4. ***p < 0.001, by one‐way ANOVA (mean ± S.E.M). B) Representative photographs of iWAT and eWAT from sham and MCAO mice at post‐stroke day 3. Scale bar: 1 cm. C) Representative images of Hematoxylin and Eosin (H&E) stained eWAT cross sections. Scale bar: 75 µm. D) The distribution of adipocyte size in eWAT at 3 days after stroke. n = 5. *p < 0.05, ***p < 0.001, in MCAO mice versus sham mice, by two‐way ANOVA (mean ± S.E.M). E) Respiratory exchange ratios of mice 1 days after stroke onset. n = 3 for sham, n = 5 for MCAO. *p < 0.05, compared with sham mice by unpaired t‐test (mean ± S.E.M). F) Genes that significantly upregulated (log2 fold change>2) in both iWAT and eWAT of MCAO mice versus sham mice were selected for analysis. Pairwise Venn diagram plots of overlapping differentially expressed genes between the iWAT and eWAT at 3 days after stroke. G) Enrichment analysis of overlapping upregulated genes between the iWAT and eWAT. H) GSEA results highlighted upregulation of long chain fatty acid metabolism in eWAT of MCAO mice compared with sham‐operated mice. I–K) Western Blot images and quantification of the expression levels of phospho‐HSL, HSL and ATGL in adipose tissues 3 days after stroke. n = 3. *p < 0.05, **p<0.01, ***p<0.001, by unpaired t‐test (mean ± S.E.M). L) IHC images showing increased UCP1 expression in eWAT after stroke. Scale bar: 25 µm. n = 5, **p<0.01, by unpaired t‐test (mean ± S.E.M). M) Quantification of adipose browning gene expression levels in eWAT of sham‐operated and MCAO mice using qRT‐PCR. Relative expression levels of the genes of interest were normalized to the housekeeping gene Hprt1. n = 4. **p < 0.01, ***p<0.001, by unpaired t‐test (mean ± S.E.M).

To uncover the mechanistic basis for adiposity‐mediated neuroprotection, we first conducted experimental investigations to examine the alterations in adipose tissue and lipid kinetics in response to stroke. Normalized weights of two major WAT depots, inguinal (iWAT) and epididymal WAT (eWAT), dropped dramatically as early as 1 day after MCAO, and declined steadily at least until the end of subacute phase of experimental stroke (3 days after MCAO) (**Figure** **1**A). Gross observation revealed that WAT depots shrunk in size and turned brown in appearance, implicating potential acute lipolysis and brown remodeling of adipose tissue in response to stroke (Figure 1B). Data from histological analyses further confirmed a gradual reduction in adipocyte size following MCAO (Figure 1C,D; Figure S1A,B, Supporting Information). Following this line, we proceeded to measure the post‐stroke energy metabolism using indirect calorimetry (IC). Under a fed‐pair condition, a reduced respiratory exchange ratio was observed 1 day after MCAO when compared to sham‐operated mice, indicative of a stroke‐induced shift towards FAs metabolism (Figure 1E; Figure S1C, Supporting Information). Meanwhile, MCAO mice exhibited a trend of compromised locomotor activity and reduced energy expenditure due to stroke paralysis (Figure S1D,E, Supporting Information). To gain insight into the underlying molecular mechanisms, we applied bulk RNA‐seq analysis of iWAT and eWAT freshly isolated from sham‐operated and MCAO mice. Gene Ontology (GO) analysis and gene set enrichment analysis (GSEA) of differentially expressed genes (DEGs) converged to suggest remarkable changes in lipid catabolism, especially triglyceride (TG) lipolysis and FA metabolism (Figure 1F,H; Figure S1F,G, Supporting Information). Western blotting analysis revealed pronounced increases in the protein levels of key enzymes responsible for TG hydrolysis, adipose triglyceride lipase (ATGL) and hormone‐sensitive lipase (HSL), after stroke (Figure 1I–K). Moreover, a substantial induction of browning markers, UCP1 and ELOVL3, was detected in WAT by immunohistochemical staining and qRT‐PCR at 3 days after MCAO (Figure 1L,M; Figure S1H,I, Supporting Information). Furthermore, we applied the specific ATGL inhibitor atglistatin (10 mg k^−1^g) to interrupt lipolysis. As expected, atglistatin significantly increase the size of WAT and reduced the expression levels of UCP1 (Figure S2A–G, Supporting Information). Interestingly, atglistatin pronouncedly potentiated neurological deficits and increased overall mortality of MCAO mice (Figure S2H,I, Supporting Information). Collectively, these data provide evidence that WAT depots undergo lipolysis and browning remodeling in the early injury phase following stroke, which potentially facilitates early stroke rehabilitation.

### 2.2 Hepatic FAO and Subsequent Ketogenesis are Activated in Ischemic Stroke

**Figure 2:**
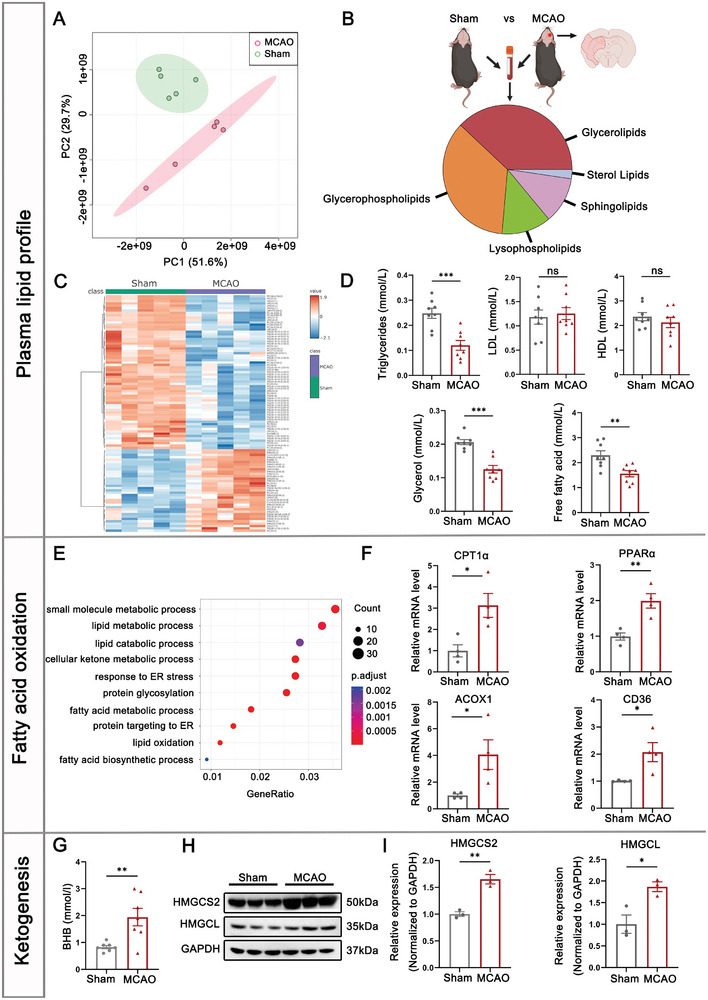
Hepatic FAO and subsequent ketogenesis are activated in ischemic stroke. A) Principal component (PC) analysis plots for non‐targeted metabolomics data. Analysis was performed on plasma collected from sham‐operated and MCAO mice 3 days after stroke. n = 5. B) Classification of plasma metabolites that were significantly altered by stroke. C) Heatmap of the top 100 differentially expressed metabolites showed significantly downregulated triglycerides in the plasma of MCAO mice. n = 5. D) ELISA assay was used to measure the plasma lipid profiles of sham and MCAO mice, including triglycerides, glycerol, free fatty acids, HDL and LDL. n = 8. **p < 0.01, ***p<0.001, by unpaired t‐test (mean ± S.E.M). E) Enrichment analysis of differentially expressed genes in the liver of MCAO mice compared with sham mice. n = 3. F) mRNA expression levels of hepatic FAO‐related genes were measured by qRT‐PCR 3 days after stroke. n = 4. *p < 0.05, **p<0.01, by unpaired t‐test (mean ± S.E.M). G) Ketone body concentrations in peripheral blood isolated from sham and MCAO mice. n = 7. **p<0.01, by unpaired t‐test (mean ± S.E.M). H) Western blot analysis revealed the elevated protein levels of HMGCS2 and HMGCL in hepatic tissue after stroke. I) Quantification of ketogenic proteins expression in liver from sham and MCAO mice. n = 3. *p < 0.05, **p<0.01, compared with sham mice by unpaired t‐test (mean ± S.E.M).

To comprehensively understand the early systemic metabolic response to stroke, we investigated the plasma lipid profiles of MCAO mice using liquid chromatography coupled–mass spectrometry (LC–MS) for untargeted lipidomics analysis. Impressively, experimental stroke mice underwent metabolic reprogramming after cerebral ischemia, resulting in global alterations in the plasma lipidome (**Figure** **2**A). We found that the majority of lipid species, especially glycerolipids (i.e., triglycerides, TG) and glycerophospholipids, were significantly altered at 3 days after stroke (Figure 2B). The Top 100 differentially expressed metabolites in MCAO mice were mainly various types of triglycerides, exhibiting a decreased tendency (Figure 2C). Consistent with lipidomic profiling, results from metabolite assays revealed a reduction in circulating TG, but no significant difference in low‐density lipoprotein cholesterol (LDL) or high‐density lipoprotein cholesterol (HDL) levels (Figure 2D). Since TG hydrolysis released free FAs and glycerol, we proceeded to assess the plasma concentration of these metabolites. Intriguingly, levels of circulating glycerol and free FAs were markedly decreased in MCAO mice, indicating peripheral uptake and utilization of these molecules after stroke (Figure 2D). Elevation in mRNA expression of central players in gluconeogenesis (PCX, PCK1 and GK) also confirmed the further metabolism of glycerol released from TGs (Figure S3A, Supporting Information).

Given the prominent role of the liver in systemic metabolism, we compared the transcriptional profiles between sham‐operated and MCAO mice. Noteworthy, unbiased GO analyses showed that FA metabolism and ketogenic pathway were pronouncedly enriched in the hepatic tissue of MCAO group (Figure 2E; Figure S3B,C, Supporting Information). Using qRT‐PCR assays, we detected elevated mRNA expression of FA transporter CD36 and key mediators in FA β‐oxidation, including CPT1α, PPARα and ACOX1, confirming a hepatic shift towards enhanced FAO following stroke (Figure 2F). High rate of FAO generates derivative acetyl‐CoA that fuels ketogenesis and other metabolic processes.^[^ ^25^ ^]^ As the rate‐limiting enzyme of ketogenesis, hydroxy‐3‐Methyl‐CoA synthase 2 (HMGCS2) catalyzes the formation of HMG‐CoA from acetoacetyl‐CoA and acetyl‐CoA. Subsequently, HMG‐CoA lyase (HMGCL) cleaves HMG‐CoA and releases acetoacetate (AcAc), which is further reduced to BHB by D‐3‐hydroxybutyrate dehydrogenase 1 (BDH1).^[^ ^26^ ^]^ As expected, we observed plasma KBs elevated by 3‐ to 4‐fold 3 days after MCAO (Figure 2G). MCAO mice displayed increased protein and mRNA expression of ketogenic enzymes (HMGCS2 and HMGCL) in their hepatic tissue (Figure 2H,I; Figure S3D, Supporting Information). Considering that inadequate food intake may contribute to ketogenesis, we provided additional nutrition support as previous study reported.^[^ ^27^ ^]^ Impressively, we found that additional nutrition support mildly suppressed ketogenesis in MCAO mice, but the levels were still higher than those in sham‐operated mice (Figure S4A,B, Supporting Information). These results suggest that stroke induces early activation of hepatic FAO and ketogenesis, while their pathophysiological implications for neurological recovery are not unequivocally clear.

### 2.3 BHB Supplementation Confers Protection against Stroke through Preserving BBB Integrity

**Figure 3:**
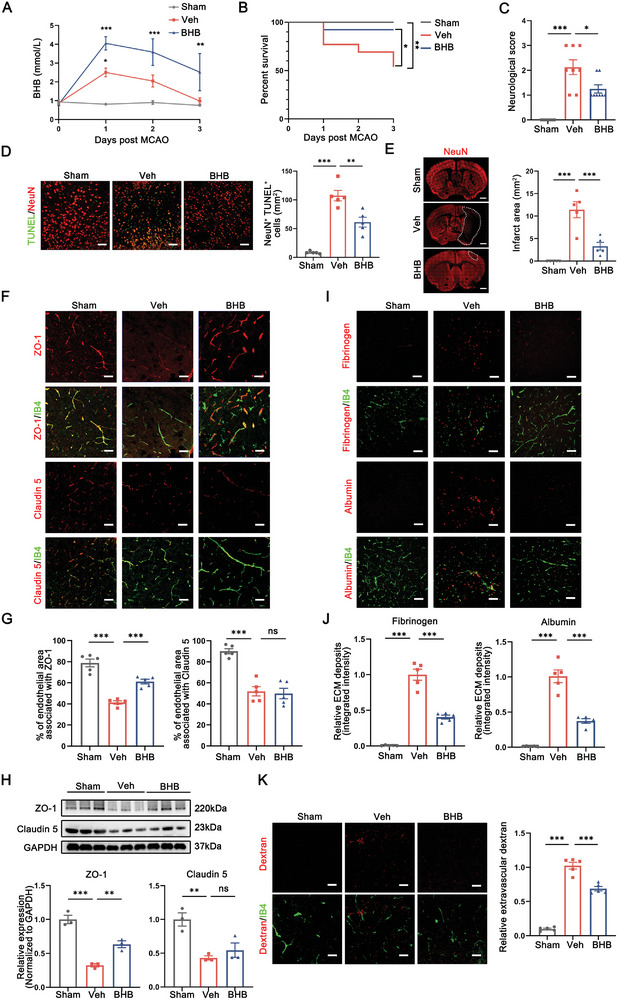
BHB supplementation confers protection against stroke through preserving BBB integrity. A) Temporal dynamics of BHB concentrations were measured after the transplantation of osmotic pumps. n = 6. **p<0.01, ***p<0.001, compared with sham mice by two‐way ANOVA (mean ± S.E.M). B) Survival curve showing the mortality rate within 3 days after stroke of each group of mice. n = 13. **p < 0.01, in sham versus vehicle‐treated group; *p < 0.05 in BHB‐ versus vehicle‐treated group, by log‐rank test. C) Neurological deficits scores were assessed during the first 3 days after MCAO. n = 8. ***p < 0.001, in sham versus vehicle‐treated group; *p < 0.05 in BHB‐ versus vehicle‐treated group, by one‐way ANOVA (mean ± S.E.M). D) Representative images and quantification analysis of TUNEL^+^ apoptotic neuron in infarct penumbra 3 days after MCAO. n = 5. **p < 0.01, ***p < 0.001, by one‐way ANOVA (mean ± S.E.M). E) Infarct area of male mice was quantified by immunostaining of NeuN. Dashed lines outline the infarct area. n = 5. ***p < 0.001, by one‐way ANOVA (mean ± S.E.M). F,G) Immunofluorescent staining for blood vessels (isolectinB4, IB4) and tight junction markers, including ZO‐1 and Claudin 5, in the peri‐infarct region of the ipsilateral hemisphere (F). Scale bar: 50 µm. Quantification of tight junction was assessed as the percentage of blood vessel area associated with ZO‐1/Claudin 5 (G). n = 5. ***p < 0.001, by one‐way ANOVA (mean ± S.E.M). H) Immunoblot analysis and quantification of BBB tight junction proteins from the ischemic hemisphere indicated that the expression levels of ZO‐1 were elevated after BHB treatment. n = 3. **p < 0.01, ***p < 0.001, by one‐way ANOVA (mean ± S.E.M). I,J) Representative images (I) and quantification analysis (J) of the extravascular albumin and fibrinogen expression surrounding IB4‐labeled blood vessels. Scale bar: 50 µm. n = 5. ***p < 0.001, by one‐way ANOVA (mean ± S.E.M). K) BBB permeability was assessed by infusion of dextran‐TRITC in sham and MCAO mice at 3 days after MCAO. Representative images showing the extravascular dextran. Scale bar: 30 µm. n = 5. ***p < 0.001, by one‐way ANOVA (mean ± S.E.M).

Recent research indicates that KBs exert a broad range of beneficial effects in the treatment of neurological disorders, such as epilepsy, Parkinson's Disease and age‐related cognitive deficits.^[^ ^28^ ^]^ In light of this evidence, we hypothesized that KBs could provide early protection against ischemic brain rather than being mere by‐products of FAs breakdown, which partially mediated the favorable role of adiposity in post‐stroke functional recovery. To verify this hypothesis, we implanted subcutaneous osmotic pumps to provide supplemental delivery of either the most abundant type of KBs, BHB (200 mg), or an equivalent volume of vehicle (Veh) in MCAO mice for consecutive 3 days (**Figure** **3**A). Remarkably, BHB treatment significantly increased the survival rate of experimental mice, with 12 of 13 (92.31%) surviving in the BHB‐treated group compared to only 7 of 13 (53.85%) in the Veh‐treated control group during the 3‐day intervention period (Figure 3B). In addition, BHB‐treated mice exhibited less severe neurological deficits as well as fewer dead or dying neurons (NeuN^+^TUNEL^+^ cells) than Veh‐treated controls (Figure 3C–E), highlighting BHB as a critical metabolite that restricted ischemic cerebral injury from an early stage.

The BBB is rapidly disrupted and increases in permeability in response to ischemic insult, leading to peripheral leukocytes infiltration and poor prognosis.^[^ ^29^ ^]^ Endothelial cells (ECs), sealed paracellularly by tight junction (TJ) proteins like Zona Occludens‐1 (ZO‐1) and Claudin 5, are pivotal constituents of BBB and an early intervention target for ischemic stroke.^[^ ^30^ ^]^ To investigate whether BHB administration improved post‐stroke recovery in a BBB‐protective manner, we examined the distribution and expression of TJ‐associated proteins 3 days after MCAO using immunostaining and western blotting. We found that BHB therapy led to upregulated expression of ZO‐1, but not Claudin 5, in IB4‐labeled ECs as compared to the control group (Figure 3F,G). In line with the results from immunostaining, western blot analysis confirmed the preserved expression of ZO‐1 after BHB treatment, while the levels of Claudin 5 showed marginal changes (Figure 3H). To gain further insights into the impacts of BHB on barrier function, we imaged the leakage of plasma proteins (i.e., fibrinogen and albumin) and a fluorescent tracer (dextran‐TRITC) across the compromised endothelium into the cerebral parenchyma. We demonstrated that early administration of BHB drastically attenuated stroke‐induced BBB dysfunction as quantified by vascular extravasation (Figure 3I–K).

### 2.4 BHB‐Mediated BBB Maintenance Engenders an Inflammation‐Resolving Phenotype in the Ischemic Brain

**Figure 4:**
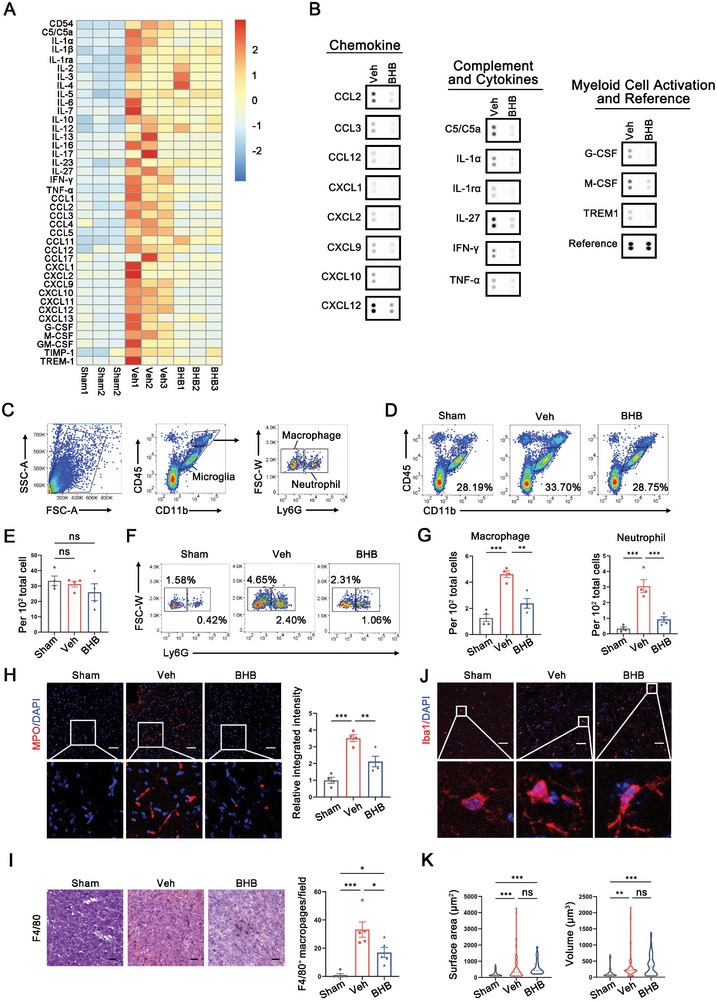
BHB‐mediated BBB maintenance engenders an inflammation‐resolving phenotype in the ischemic brain. A,B) Heatmap (A) and representative dot plots (B) showing the expression levels of inflammatory factors including chemokines, cytokines, complements and myeloid cell activators in peripheral blood after ketone body treatment. The reference spots served as positive controls to normalize the greyscale density of tested cytokines. n = 3. C) Gating strategy for brain‐resident macrophages (CD11b^+^CD45^hi^Ly6G^−^), neutrophils (CD11b^+^ CD45^hi^Ly6G^+^) and microglia (CD45^int^CD11b^+^). (D‐G) FACS analysis D, F) and quantification E, G) for the proportion of microglia, neutrophils and macrophages in the ipsilateral hemisphere after BHB administration. n = 4. **p < 0.01, ***p < 0.001, by one‐way ANOVA (mean ± S.E.M). H–I) Representative staining and quantification of neutrophils (MPO^+^, red, G, n = 4) and macrophages (F4/80^+^, H, n = 5) in the peri‐infarct region at 3 days after stroke. Scale bar: 75 µm. *p < 0.05, **p<0.01, ***p < 0.001, by one‐way ANOVA (mean ± S.E.M). J) Representative images demonstrating microglia/macrophage marker Iba1^+^ in the ipsilateral hemisphere 3 days following stroke. Scale bar: 50 µm. K) Statistical analysis of the surface area and volume of microglia. n = 100 cells from four experimental mice for morphology analysis. **p < 0.01, ***p < 0.001, by one‐way ANOVA (mean ± S.E.M).

Given that BBB breakdown potentiates post‐stroke leukocyte infiltration and aggravates local neuroinflammatory cascade,^[^ ^31^ ^]^ we sought to address the effects of BHB on immune milieu in the ischemic brain. Brain extracts were examined for a panel of inflammatory factors using a commercial protein array 3 days after MCAO. Post‐stroke BHB therapy resulted in a substantial decrease in the expression of proinflammatory chemokines (CCL3, CXCL1, CXCL9, CXCL10), complement (C5/5a), cytokines (IFN‐γ), and myeloid cell activators (G‐CSF) in the ischemic hemisphere (**Figure** **4**A,B; Figure S5A,B, Supporting Information). Thereafter, we assessed potential impacts of BHB treatment on immune cell infiltration. During the first 3 days of stroke, neutrophils are among the first immune cells to flood into the ischemic lesion, followed by macrophage migration that peaks around 2–3 days after stroke.^[^ ^32^ ^]^ Flow cytometry analysis revealed that BHB treatment preeminently decreased the counts of myeloid cells in stroke lesions, whereas had little influence on the number of resident microglia (Figure 4C–E). The cell counts of both neutrophils and macrophages were markedly diminished after systemic BHB administration for 3 days (Figure 4F,G). Consistently, immunostaining for neutrophil activation marker myeloperoxidase (MPO) and microglia/macrophage marker F4/80 confirmed the lower immune infiltration phenotype in ischemic brains of BHB‐treated mice (Figure 4H,I). However, the activation of microglia/macrophages, characterized by enlarged cell body and ramified morphology, was not significantly blunted by BHB administration (Figure 4J,K). Overall, these observations provide novel insights into the protective role of post‐stroke ketogenesis, which reinforces the structural and functional integrity of BBB and alleviates the ensuing neuroinflammatory responses.

### 2.5 Blocking FAs Mobilization and Hepatic Ketogenesis Exacerbates BBB Disruption and Aggravates Stroke Progression

**Figure 5:**
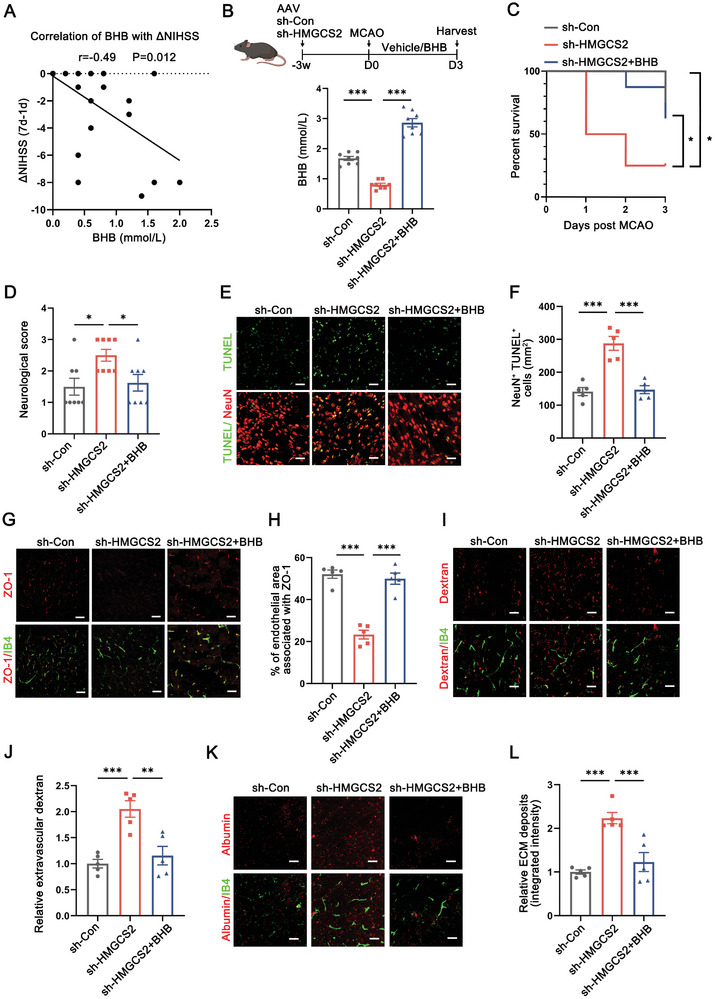
Blocking hepatic ketogenesis exacerbates BBB disruption and aggravates stroke progression. A) Correlation between delta NIHSS (NIHSS at 7d minus NIHSS at 1d) with plasma BHB concentration was estimated with Spearman correlation analysis. n = 26. B) AAV expressing sh‐HMGCS2 or sh‐Con was i.v. injected into the experimental mice three weeks prior to MCAO. Mice of sh‐HMGCS2+BHB group received BHB administration after stroke for 3 days. Serum BHB levels were detected to verify knockdown efficiency. n = 8. ***p < 0.001, by one‐way ANOVA (mean ± S.E.M). C) Survival curves showing the mortality rate within 3d after stroke in sh‐Con and sh‐HMGCS2‐treated MCAO mice with or without BHB injection. n = 8. *p < 0.05, compared with sh‐HMGCS2 ‐treated group by log‐rank test. D) Neurological deficit scores were evaluated in the indicated group of experimental mice. n = 8. *p < 0.05, by one‐way ANOVA (mean ± S.E.M). E,F) Representative images (E) and quantification analysis (F) of TUNEL^+^ apoptotic neuron in the infarct penumbra of MCAO mice. Scale bar: 50 µm. n = 5. ***p < 0.001, by one‐way ANOVA (mean ± S.E.M). G,H) Immunofluorescent staining for blood vessels (isolectinB4, IB4) and tight junction marker (ZO‐1) in the peri‐infarct region of the ipsilateral hemisphere. Scale bar: 50 µm. n = 5. ***p < 0.001, by one‐way ANOVA (mean ± S.E.M). I–L) Representative confocal images and quantification analysis of the extravascular dextran (I,J) and albumin (K,L) deposition around IB4‐labeled endothelium. Scale bar: 30 µm. n = 5. **p<0.01, ***p < 0.001, by one‐way ANOVA (mean ± S.E.M).

Following this line, we continued to determine the pathophysiological implications of adaptive FAO‐ketogenesis processes in early neuroprotection after stroke. First, we found that plasma BHB levels (acute phase, within 1d after disease onset) were negatively correlated with neurological recovery indicated by delta NIHSS (7d‐1d) in stroke patients, suggesting that adaptive ketogenesis could impede stroke progression (**Figure** **5**A). To explore the impacts of blocking FAO‐ketogenesis processes on stroke severity, we applied the antagonist of CPT1α etomoxir (20 mg k^−1^g) to interrupt the rate‐limiting step of FAO and partially eliminate stroke‐induced BHB elevations (Figure S6A, Supporting Information). Interestingly, etomoxir pronouncedly potentiated neuronal death, neurological deficits and increased overall mortality of MCAO mice (Figure S6B–D, Supporting Information). Immunostaining of brain slices from etomoxir‐treated mice revealed a progressive loss of ZO‐1 expression and substantially increase of extravascular albumin and dextran‐TRITC deposition as compared to Veh‐treated controls (Figure S6E–G, Supporting Information). To be noted, such aggravated neurological deterioration and disrupted BBB integrity could be partially restored by BHB supplementation.

To further consolidate that adaptive ketogenesis is vital to stroke prognosis, we systematically delivered adeno‐associated virus serotype DJ (AAV‐DJ) carrying short‐hairpin RNA targeting HMGCS2 (sh‐HMGCS2) or scrambled shRNA as a control (sh‐Con) 3 weeks prior to MCAO surgery. The liver was specifically infected by AAV‐DJ, and the confocal fluorescence images showed that approximately 60% hepatocytes were GFP‐positive (Figure S7A, Supporting Information). The knockdown efficiency was verified by qRT‐PCR analysis (Figure S7B, Supporting Information). As expected, knockdown of HMGCS2 resulted in decreased circulating BHB levels after stroke (Figure 5B; Figure S7C, Supporting Information). Dampened HMGCS2‐mediated ketogenesis markedly increased overall experimental mice and neuronal death following MCAO (Figure 5C–F). In compliance with the findings from etomoxir treatment, AAV‐DJ‐sh‐HMGCS2 exacerbated BBB leakage, facilitated fluorescent dye extravasation, and promoted disease progression, which could be partially rescued by BHB infusion (Figure 5G–L). In aggregate, these findings established BHB as a crucial executor of adaptive lipid metabolism that confers early neuroprotection against stroke.

### 2.6 Inability to Transport BHB via MCT1 in Endothelial Cells Compromises the Therapeutic Efficacy of Early Ketotherapy

**Figure 6:**
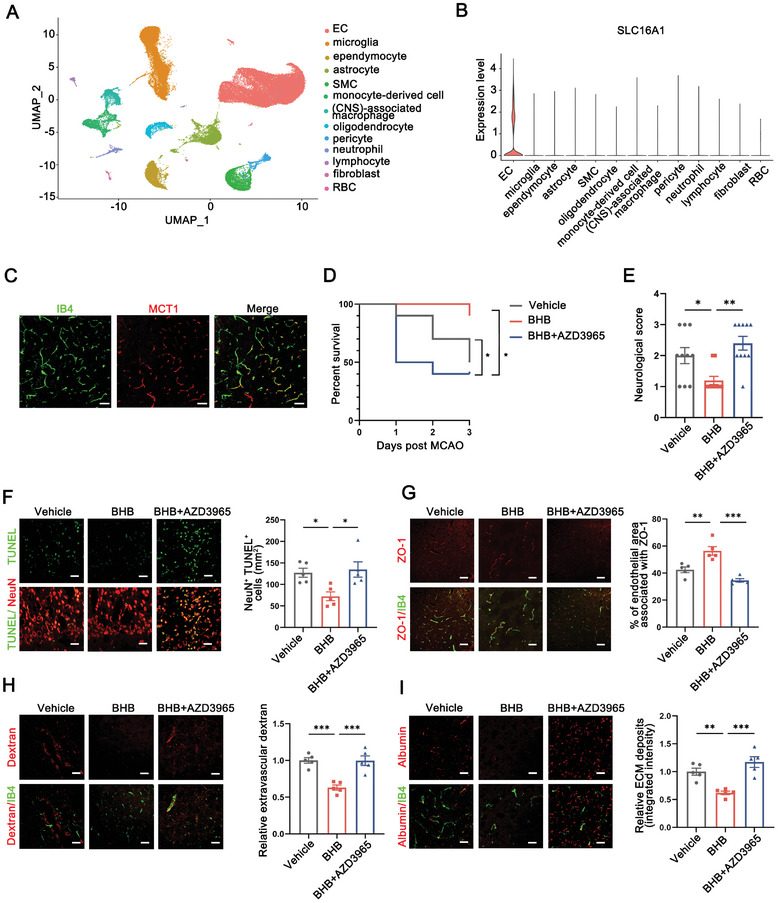
Inability to transport BHB via MCT1 in endothelial cells compromises the therapeutic efficacy of early ketotherapy. A) Uniform manifold approximation and projection (UMAP) plot of brain single‐cell sequencing data. B) Violin plot of SLC16A1 (encoding MCT1) transcript expression in EC. C) Immunofluorescent staining indicated co‐localization of MCT1 and IB4‐labeled ECs. Scale bar: 50 µm. D) Survival curves depicted the mortality rate within 3 days after stroke in mice treated with either vehicle or BHB, with or without AZD3965 administration. n = 10. *p < 0.05, by log‐rank test. E) Each group of mice underwent evaluation for neurological deficit scores. n = 10. *p < 0.05, **p<0.01, by one‐way ANOVA (mean ± S.E.M). F) Representative confocal images and quantitative analysis highlight TUNEL^+^ apoptotic neurons in the infarct penumbra of mice in our experiment after stroke. Scale bar: 50 µm. n = 5. *p < 0.05, by one‐way ANOVA (mean ± S.E.M). G) Immunostaining for blood vessels (IB4) and tight junctions (ZO‐1) in the peri‐infarct region of the ipsilateral hemisphere. Scale bar: 50 µm. n = 5. **p<0.01, ***p < 0.001, by one‐way ANOVA (mean ± S.E.M). H,I) Representative confocal images and analyses quantifying dextran (H) and albumin (I) extravasation around IB4‐labeled blood vessels. Scale bar: 30 µm. n = 5. ***p < 0.001, by one‐way ANOVA (mean ± S.E.M).

A remaining question is to comprehensively understand the cellular and molecular mechanisms by which BHB maintained BBB integrity after stroke. Utilizing a cerebral single‐cell sequencing dataset released by Kai et al.,^[^ ^33^ ^]^ we found that monocarboxylic acid transporter 1 (MCT1) responsible for KBs transport, encoded by gene SLC16A1, was exclusively expressed in ECs rather than other components of BBB.^[^ ^33^ ^]^ (**Figure** **6**A,B). Immunostaining further corroborated the expression of MCT1 in IB4^+^ ECs, implicating the endothelium as a potential cellular target of BHB (Figure 6C). To further exclude the possibility that BHB function on BBB depend on other factors (i.e., orchestrating microglia and alleviating neuroinflammation), we treated MCAO mice with the MCT1 inhibitor AZD3965 (20 mg k^−1^g, orally gavage) in combination with BHB to block the uptake of BHB by ECs. Remarkably, we found that the administration of AZD3965 significantly hampered the therapeutic effects of BHB on BBB preservation, neuroprotection, and stroke rehabilitation (Figure 6D–I). These findings suggest that BHB functions on BBB primarily in an endothelium‐dependent manner, whereas the underlying regulatory mechanisms remain to be elucidated.

### 2.7 BHB Upregulates ZO‐1 Expression in Endothelial Cells through Epigenetic Modification of H3K9 β‐hydroxybutyrylation (H3K9bhb)

**Figure 7:**
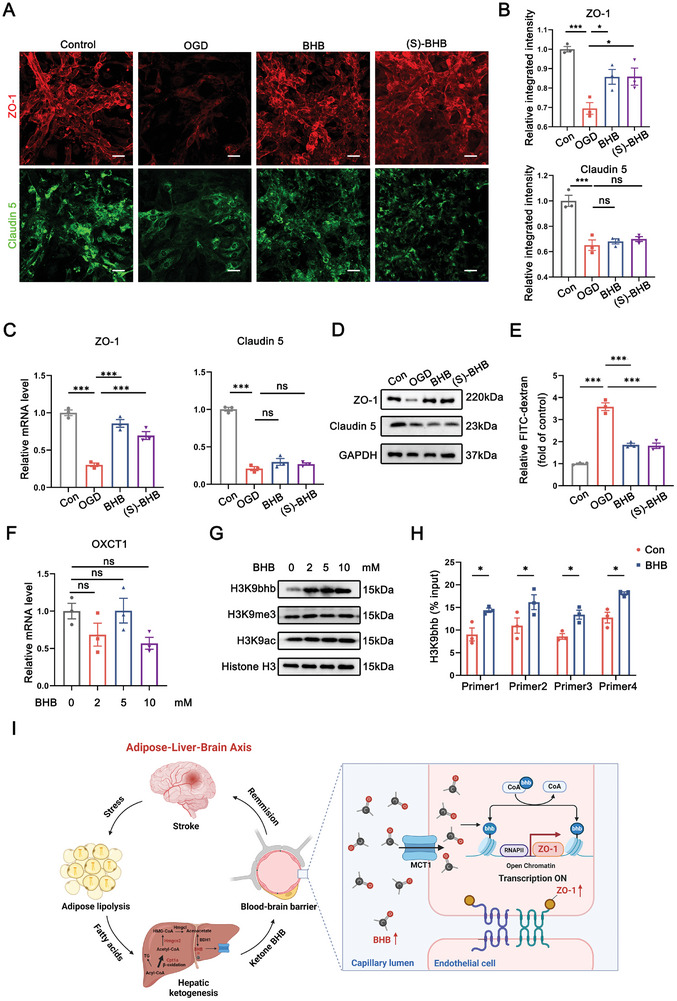
BHB upregulates ZO‐1 expression in endothelial cells through the epigenetic modification of H3K9bhb. A,B) Immunofluorescent staining of ZO‐1 and Claudin 5 in BHB or (s)‐BHB‐treated bEnd.3 cells (A). Scale bar: 50 µm. The mean fluorescence intensity (MFI) of tight junction proteins was quantified in randomly selected microscopic fields from triplicates (B). n = 3. *p<0.05, ***p < 0.001, by one‐way ANOVA (mean ± S.E.M). C) mRNA expression levels of tight junction‐related genes were measured by qRT‐PCR in cultured bEnd.3 cell. n = 3. ***p < 0.001, by one‐way ANOVA (mean ± S.E.M). D) Western blot analysis of endothelial tight junction protein expression, including ZO‐1 and Claudin 5, in cultured bEnd.3 cells treated with BHB and (s)‐BHB. Data was collected from three independent experiments. E) Transcytosis of FITC‐labeled 10‐kDa dextran across bEnd.3 cells after BHB treatment for 1 day. n = 3. ***p < 0.001, by one‐way ANOVA (mean ± S.E.M). F) Quantification of mRNA expression of ketolysis gene OXCT1 measured by qRT‐PCR in cultured bEnd.3 cell. n = 3. by one‐way ANOVA (mean ± S.E.M). G) Western blot analysis for H3K9bhb, H3K9‐acetylation (H3K9ac) and H3K9‐methylation (H3K9me3) from each group of bEnd.3 cells. H) ChIP‐qPCR analysis of H3K9bhb occupancy at the promoter of TJP1 (ZO‐1) gene in the indicated group. n = 3. *p<0.05, by two‐way ANOVA (mean ± S.E.M). I) Schematic diagram displaying that adipose tissue protects BBB function during stroke via fueling ketogenesis.

Furthermore, BHB has been established to function preferentially as an alternative metabolic fuel or epigenetic modifier in different pathological contexts.^[^ ^2^, ^21^, ^34^ ^]^ To identify the predominant pathway, S‐BHB, an enantiomer of BHB that cannot enter the Krebs cycle as an energy substrate,^[^ ^35^ ^]^ was applied to treat murine cerebral endothelium cell line bEnd.3 cells. Of note, we found that S‐BHB enhanced protein and mRNA expression of ZO‐1, but had minimal influence on Claudin 5 levels, similar to the effects of D‐BHB in OGD‐treated ECs (**Figure** **7**A–D). In compliance with the in vivo data shown in Figure 3, S‐BHB exhibited a barrier protective phenotype comparable to D‐BHB in cultured ECs (Figure 7E). Moreover, the expression of 3‐oxoacid CoA‐transferase 1 (OXCT1), a mitochondrial enzyme responsible for ketone utilization, showed marginal alterations after BHB treatment, implying a low likelihood of enhanced ketone catabolic pathway (Figure 7F). We also assessed mitochondrial superoxide (mtROS) and mitochondrial membrane potential (MMP) using mitoSOX and tetramethylrhodamine (TMRM), respectively. FACS results illustrated that the protective effects of BHB on OGD‐treated ECs were independent of the modulation of mitochondrial homeostasis (Figure S8A–D, Supporting Information). Considering these results, we preferentially speculated BHB as a signaling mediator rather than an alternative energy substrate in this scenario.

To further decipher the epigenetic mechanism by which BHB upregulated ZO‐1 expression (encoded by the TJP1 gene), we examined canonical histone modification sites, including acetylation, methylation and β‐hydroxybutyrylation of histone H3. Immunoblotting revealed a specific elevation in H3K9bhb after BHB treatment, while the levels of H3K9me3 and H3K9ac were almost unaffected (Figure 7G; Figure S9A, Supporting Information). Treatment with HDAC inhibitor trichostatin A (TSA) or MS‐275 failed to rescue ZO‐1 expression in bEnd.3 cells, further ruling out the possibility of BHB acting as a deacetylase inhibitor under this scenario (Figure S9B, Supporting Information). Additionally, chromatin immunoprecipitation (ChIP) coupled with qRCR analysis on the gene TJP1 using four distinct primer pairs targeting different genomic locations revealed an enrichment of H3K9bhb in the TJP1 promoter region following high‐dose BHB treatment (Figure 7H). In summary, these results suggest that BHB improves barrier function by epigenetically modifying TPJ1‐bound H3K9. As such, we propose a novel adaptive metabolic mechanism that mediates early BBB preservation after stroke, laying the groundwork toward promising strategies targeting metabolic responses to treat stroke patients.

## 3 Discussion

Favorable prognosis and survival advantage have been observed in overweight/obese individuals with various critical illness, especially cardiovascular and cerebrovascular diseases, which is termed as obesity paradox.^[^ ^22^, ^36^ ^]^ In the present study, we experimentally delineate a fundamental metabolic mechanism whereby adaptive lipolysis stimulates FAO‐ketogenesis processes to counteract acute ischemic brain injury. Mechanistic studies highlight BHB as a pivotal signaling metabolite that facilitates BBB preservation and subsequent inflammation infiltration via facilitating β‐hydroxybutyrylation of tight junction gene TJP1 (Figure 7I). We believe these findings provide promising approaches for early intervention in stroke patients by targeting adaptive metabolic responses.

Acute critical illness, such as stroke, elicits a cascade of metabolic adaptions characterized by substantially increased energy expenditure and whole‐body catabolism to optimize nutrient utilization and drive host survival.^[^ ^37^ ^]^ As a highly dynamic organ, adipose tissue undergoes lipolysis and releases lipid signals in response to adrenergic stress and energy deficits following critical illness.^[^ ^38^ ^]^ In the present study, we performed LC‐MS for unbiased lipidomic analysis and unraveled a dramatic decrease in the levels of major lipid species, especially TG, during the acute phase of stroke. These findings were consistent with recent studies on critical illness, including acute trauma and COVID‐19,^[^ ^39^ ^]^ suggesting that altered lipidomic patterns might be generalized to severe illness resulting from diverse etiologies. Furthermore, emerging evidence correlated this global lipidome and metabolome remodeling with the clinical evolution of critically ill patients.^[^ ^2^, ^39^ ^]^ Abdullahi et al. have illustrated that impaired brown remodeling and oxidative capacity in WAT is significantly associated with reduced survival rate in elderly patients with severe burn trauma.^[^ ^2a^ ^]^ Moreover, preserved circulating lipid levels have been implicated to be responsible for the survival benefit of early thawed plasma administration.^[^ ^39a^ ^]^ Collectively, these lines of evidence indicate that the increased availability of lipids released by acute triglyceride lipolysis might account for the protective impacts of adiposity against critical illness,^[^ ^40^ ^]^ whilst the downstream mechanisms are not unequivocally clear.

Conventional view posits that the liver functions as a pivotal metabolic hub for maintaining the metabolic homeostasis of energy substrates, including carbohydrates, amino acids, and FAs.^[^ ^14^ ^]^ Adipocyte lipolysis‐derived FAs are delivered to the liver and serve as critical regulators of hepatic gene expression and metabolic responses to nutritional challenges, indicating an adipose to liver dialogue.^[^ ^41^ ^]^ Nevertheless, knowledge about alterations of the adipose tissue‐liver axis during stroke progression and how this crosstalk in turn affects neurological recovery remains lacking. Herein, we detected enhanced CPT1α‐mediated FAO and HMGCS2‐induced hepatic ketogenesis processes in experimental stroke using transcriptomic profiling and metabolite assays. Accordingly, blockage of FAO‐ketogenesis processes by CPT1α antagonism or HMGCS2 knockdown aggravated the cerebral damage and exacerbated stroke severity. Additionally, plasma BHB concentrations were found to be positively associated with ΔNIHSS of stroke patients. These findings unveil that endogenous hepatic FAO‐ketogenesis functions as an adaptive metabolic switch to facilitate early post‐stroke rehabilitation, implying the potential therapeutic value of manipulating ketone metabolism for stroke patients.

While FAO promotes hepatic ketogenesis by generating derivative acetyl‐CoA, KBs flow down the concentration gradient through transmembrane transporter MCT1 into extrahepatic tissues such as skeletal muscle, heart, and brain.^[^ ^42^ ^]^ Unlike the glucose utilization modulated by neuronal activity, the rate of brain uptake of KBs is directly proportional to upregulated hepatic ketone production and plasma ketone concentrations.^[^ ^16^ ^]^ Given its function as an ancillary substrate and signaling mediator, BHB has been reported to exert pleiotropic effects in the brain under both physiological and pathological conditions.^[^ ^25^ ^]^ Previous neuron‐centric studies have shown that KBs coordinate neuronal excitability and neurotransmitter release by altering gene expression profiles and oxidative stress responses.^[^ ^43^ ^]^ In this study, we revealed for the first time that exogenous BHB supplementation substantially attenuated BBB permeability and ameliorated subsequent inflammatory infiltration. By virtue of their continuous exposure to circulating KBs, vascular ECs were found to exhibit improved TJ integrity and enhanced barrier function under BHB treatment. These findings were consistent with recent observations of BHB‐mediated EC protection in the cardiovascular and lymphatic systems.^[^ ^44^ ^]^ Garcia‐Caballero et al. have shown that KBs oxidation comparably stimulate lymphatic ECs replication and lymphangiogenesis by generating acetyl‐CoA to sustain tricarboxylic acid cycle (TCA) and ATP production.^[^ ^44a^ ^]^ In the heart, another major consumer of KBs, cardiac ECs exclusively metabolize KBs, thereby enhancing proliferation, migration and vessel sprouting.^[^ ^44b^ ^]^ In light of these findings, we speculate that ECs could take up KBs to sustain their structure and function during acute stress, rather than simply transporting circulating KBs to tissue cells.

Our findings underscored that BHB exerted neurovascular effects beyond their traditional role as alternative metabolic fuels for energy‐starved brain metabolism. Specifically, we delineated that provision of S‐BHB, an enantiomer of BHB that cannot enter the TCA cycle, upregulated the expression of TJ protein ZO‐1 to a similar extent as BHB treatment, suggesting that BHB‐mediated endothelial preservation was independent of chirality and terminal oxidation. Recent studies have established BHB as a novel epigenetic mark that couples metabolic alterations to gene expression. Xie et al. firstly described and identified lysine β‐hydroxybutyrylation (Kbhb) as a novel post‐translational modification in which BHB is covalently attached to lysine ε‐amino groups of core histones H3 and H4.^[^ ^22a^ ^]^ To be more specific, BHB is enzymatically added to lysine by the acetyltransferase p300 and is removed by HDAC 1 and 2.^[^ ^45^ ^]^ In response to ketogenic conditions (i.e., fasting, ketogenic diet, diabetic ketoacidosis), elevated BHB levels induces enrichment of H3K9bhb in the promotor regions of critical gene subsets, facilitating chromatin opening and target gene expression in the liver, small intestine, pancreas and immune cells.^[^ ^22^, ^46^ ^]^ Herein, we detected increased H3K9bhb binding at the promoter region of TJP1 gene in cerebral microvascular ECs treated with BHB, leading to epigenetically upregulated ZO‐1 expression. In stark contrast, BHB had only a marginal impact on the levels of histone acetylation and methylation. These findings suggest that BHB more profoundly induced histone β‐hydroxybutyrylation rather than other epigenetic modifications in OGD‐treated bEnd.3 cells. On this basis, we propose H3K9bhb modification as a novel mechanism by which BHB upregulates ZO‐1 expression and alleviates endothelial hyperpermeability in response to cerebral ischemic‐reperfusion injury.

In conclusion, our study provides evidence that adipose‐induced neuroprotection in ischemic stroke is partially mediated by enhanced lipolysis and subsequent FAO‐ketogenesis processes. Furthermore, early administration of exogenous KBs remarkably improves neurological recovery, indicating that boosting ketogenesis holds promise as an effective therapeutic strategy for stroke patients. Interventions targeting adaptive metabolic responses warrant future clinical trials. Mechanistically, our results show that BHB preserves BBB integrity and enhances endothelial barrier function by epigenetic induction of ZO‐1 expression, implicating proof‐of‐concept therapeutic potential for BHB in treating post‐stroke endotheliopathy.

## 4 Experimental Section

### Human Material

The clinical study involving stroke patient samples was approved by the ethics committee of the Third Affiliated Hospital of Sun Yat‐sen University. Peripheral blood samples were collected from 18 stroke patients recruited in the Third Affiliated Hospital of Sun Yat‐sen University within 1d after the onset of stroke. NIHSS of patients were assessed and recorded at 1d and 7d after stroke onset according to previously published guidelines.^[^ ^47^ ^]^ Plasma samples were rapidly collected by centrifugation at 15000 g for 15 min, and subjected to BHB examination using Abbott ketone strips by investigators blinded to the clinical characteristics of patients. The demographic characteristics of stroke patients were summarized in Table S1 (Supporting Information).

### Animals

In the current study, male C57BL/6 mice aged 8‐ to 12‐ week‐old were purchased from GemPharmatech Co., Ltd. The animals were kept in individually ventilated cages under specific‐pathogen‐free (SPF) conditions, with ad libitum access to water and food. The animals were housed in this humidity‐ and temperature‐controlled facility, with a 12‐hour light‐dark cycle for at least one week prior to further experiments. All animal experiments were performed under the ethical approval of the Ethical Committee of Sun Yat‐Sen University.

### Experimental Model

Transient middle cerebral artery occlusion (MCAO) was induced as previously described.^[^ ^12^ ^]^ In brief, mice were randomly assigned to either a sham or MCAO group, following anesthesia with 1.5% isoflurane in an O_2_/N_2_O mixture. A 10‐mm midline neck incision was made to further expose and isolate the right‐side common carotid artery, internal carotid artery (ICA) and external carotid artery (ECA). A monofilament with a silicone‐coated tip was inserted into ECA to block the blood supply of MCA through ICA. After 60 min of ischemia, the monofilament was withdrawn to initiate reperfusion, and the incision was carefully sutured. Sham‐operated mice underwent the same procedures as the MCAO group, including anesthesia and anatomic exposure of the arteries, except for the occlusion of MCA by filament insertion. Throughout the surgical procedure, the core temperature of all animals was maintained at 37 ± 0.5 °C with a heating pad. The neurological deficit score was assessed right after the reperfusion and every 24 hours over the next three days using a 0–4 scale as previously described:^[^ ^48^ ^]^ 0, no observable deficit; 1, weakness in the ipsilateral forelimb; 2, spontaneous circulating to the ipsilateral side (right); 3, partial paralysis on the ipsilateral side; 4, no spontaneous motor activity or death. To exclude potential impacts of food intake on metabolic measurements, sham‐operated and MCAO mice were pair‐fed after surgery. We adopted the additional nutrition support protocol utilized in the study by Lourbopolous et al.,^[^ ^27^ ^]^ and fed the additional nutrition group with 1 ml of jelly‐formed food made by ordinary mouse maintenance food and xanthan gum.

### Indirect Calorimetry (IC)

After 48 h of habituation, the experimental mice were randomly assigned to either a sham or MCAO group, while ensuring their initial body weights and food intake matched. Subsequent MCAO or sham surgery was conducted. To measure oxygen consumption, carbon dioxide production and respiratory exchange ratio (RER), we performed indirect calorimetry (IC) using a combined metabolic monitoring system (PRO‐MRM‐16 MX2 Interface 16). Each mouse in both groups was individually housed in a metabolic cage with a temperature‐controlled environment and ad libitum access to standard food and water. Experimental data was collected for 24 hours.

### Drug and Adeno‐Associated Virus (AAV) Delivery

For in vivo BHB treatment, BHB (166 898, Sigma) was dissolved in 0.9% sterile saline to achieve a final concentration of 1 g ml^−1^. The solution was then loaded into an Alzet mini‐osmotic pump (2001D), which was implanted subcutaneously on the back of mice, slightly posterior to the scapulae, immediately after reperfusion. The pump released BHB at a rate of 8 µl h^−1^ for consecutive 3 days.

MCT1 inhibitor AZD3965 (20 mg k^−1^g) or ATGL inhibitor atglistatin (10 mg k^−1^g) was dissolved in 0.5% carboxy methylcellulose (CMC) solution, delivered through oral gavage. To inhibit hepatic ketogenesis in vivo, the experimental mice received an intraperitoneal injection of CPT1α inhibitor etomoxir (20 mg k^−1^g, Sigma) or saline, one hour prior to MCAO.

AAV‐DJ‐shRNA‐GFP under the control of the albumin promoter were purchased from PackGene. To silence the hepatic HMGCS2 gene in vivo, mice were administered via tail vein with 100 µl of AAV (3×e^12^ total vector genomes) carrying either sh‐Con (a non‐targeting control vector expressing GFP) or sh‐HMGCS2 three weeks prior to MCAO. sh‐Con: 5′ TTCTCCGAACGTGTCACGTAA 3′. sh‐HMGCS2#1: 5′‐GGAAGCAAGCTGGAAACAACC‐3′sh‐HMGCS2#2:5′‐GGGAACATGTACACCTCTCTTCC‐3′. By assessing the transcription level of HMGCS2 in the liver and the circulating level of BHB, we identified sh‐HMGCS2#1 with a higher interference efficiency against HMGCS2 and utilized it for further experiments (referred to as sh‐HMGCS2).

### Non‐Targeted Metabolomics

At 3 days following stroke, plasma samples were collected from both sham and MCAO groups (n = 5/group). The harvested samples were then prepared for further analysis by mixing 50 µl of plasma with 500 µl of methanol, 150 µl of double‐distilled water (ddH_2_O), and 500 µl of chloroform, followed by vortexing for 5 minutes and centrifugation at 15 000 g for 5 min. The resulting supernatant was transferred to a 1.5 ml centrifuge tube and dried under vacuum. Before analysis, the dried extract was reconstituted with 60 µl of methanol, 30 µl of chloroform, and 10 µl of ddH_2_O, vortexed for 5 minutes, and centrifuged at 15 000 g for 5 minutes. Pooled quality control (QC) samples were also prepared by combining 75 µl aliquots of each biological sample.

The QExactive LC system (Thermo Fisher Scientific) was used to analyze samples in both positive and negative ion modes. Chromatographic separations were performed using a Hyperil Gold C18 column (100×2.1 mm, 1.9 µm, Thermo Fisher Scientific) operating at a column temperature of 40 °C. The separations were carried out at a flow rate of 300 µl per minute with two solvents, namely Solvent A = 0.1% formic acid in 6/4 acetonitrile/water and solvent B = 0.1% formic acid in 9/1 isopropanol/acetonitrile. All column eluent was transferred to the mass spectrometer, and full‐scan profiling data were acquired in the m/z range 100–1500 in the full scan/data‐dependent ms^2^ Top10 analysis. The source and ion transfer parameters applied were as follows: source heater = 320 °C, sheath gas = 40 (arbitrary units), aux gas = 10 (arbitrary units), capillary temperature = 350 °C, ISpray voltage = 3.5 kV (positive‐ion mode) and 3 kV (negative‐ion mode).

Raw data from full scan/data‐dependent ms^2^ Top10 analysis were analyzed using Lipid Search 4.1 software (v 4.1, Waters Corp.) to calculate the concentration of each analyte in the samples. Unsupervised Principal Components Analysis (PCA) and differential analysis were performed using an open access software MetaboAnalyst 5.0 (www.metaboanalyst.ca).

### RNA Sequencing and Bioinformatic Analysis

Total RNA was isolated from the liver, brain, epididymal fat and subcutaneous fat from experimental mice aged 10–12 weeks using RNeasy Micro Kit (QIAGEN) according to manufacturer's instruction. The libraries for sequencing were prepared using NuGEN Ovation System V2 RNA–Seq. Transcription into cDNA was constructed using random hexametric and poly‐T primers, and the resulting cDNA libraries were amplified using the Ultralow DR library kit (NuGEN) according to the manufacturer's instructions. The quality of cDNA libraries was analyzed by Bioanalyzer (Agilent Technologies). Next‐generation sequencing was performed using a HiSeq 2000 (Illumina) platform, and the sequenced reads were mapped to mouse reference genome mm10 using Bowtie2 (v.2.3.5).

For bioinformatics analysis, DESeq2 (v.1.34.0) was used to identify DEGs (differential expression genes) in different groups of mice. Gene set enrichment analysis (GSEA) was performed using clusterProfiler (v.4.0.5), and the Gene Ontology database was selected for enrichment analyses.

### Lipid Profile Test

After the sacrifice, 1 ml peripheral blood was collected from each experimental mouse, and subsequently centrifuged at 3000 g for 10 min to collect serum. The concentration of glycerol (ab65337, Abcam), free fatty acids (MAK044, Sigma), triglycerides (MAK266, Sigma), low‐density lipoprotein (LDL, A113‐1‐1, Nanjing Jiancheng Bioengineering Institute), high‐density lipoprotein (HDL, A112‐1‐1, Nanjing Jiancheng Bioengineering Institute) and ketone body of serum (700 190, Cayman Chemical) were tested by enzyme‐linked immunosorbent assay (ELISA) according to the manufacturer's protocol.

### Assessment of BBB Permeability

Dextran conjugated to tetramethylrhodamine isothiocyanate (dextran‐TRITC), at a concentration of 10 mg ml^−1^ in saline (0.1 ml per mouse), was intravenously administered to assess in vivo blood‐brain barrier (BBB) permeability. Three hours following the injection of dextran‐TRITC, the mice were euthanized, and their brains were expeditiously harvested. The extravasation of dextran‐TRITC was visualized through confocal microscopy and subjected to detailed quantitative analysis using ImageJ software.

### Mitochondrial Membrane Potential and ROS Assessment

bEND.3 cells were stained with the mitochondrial membrane potential (MMP) probe tetramethylrhodamine (TMRM, Thermo Fisher Scientific) at 37 °C for 30 min and analyzed by flow cytometry. To assess mitochondrial ROS levels, bEND.3cells were labeled with fluorescent probe, MitoSOX Red (Thermo Fisher Scientific), at 37 °C for 30 min, and the fluorescent intensity was determined by flow cytometry.

### Protein Extraction and Western Blotting

For protein extraction from cultured cells, cells were harvested, washed three times with ice‐cold PBS. Subsequently, the cells were lysed directly in 1× RIPA buffer, and subjected to centrifugation at 10,000 g for 10 min at 4 °C. Each supernatant was recovered as a total cell lysate. Protein extracted from liver, brain, subcutaneous fat and epididymal fat tissue was used the Minute Total Protein Extraction Kit (SD‐001/SN‐002, Invent) following the manufacturer's instructions. The total protein concentration of collected samples was assessed with BCA Protein Assay Kit (Thermo Fisher), equal amounts of protein were resolved by SDS polyacrylamide gel electrophoresis and then electrotransferred to a 0.45 µm pore‐sized polyvinylidene difluoride membrane (Millipore, Darmstadt, Germany) and blocked with a 5% bovine serum albumin (BSA) solution. Then, following primary antibodies were used for incubation with the membrane. After incubation with peroxidase‐conjugated secondary antibodies, immunoreactivity was finally semi‐quantitatively detected by ChemiDoc Imaging Systems (Bio‐Rad). The commercially available antibodies used for Western blotting were provided in Table S2 (Supporting Information).

### RNA Analysis and Quantitative PCR

Total RNA extracted from tissues or cells was carried out using the RNeasy mini kit (QIAGEN) according to the manufacturer's instructions. The purity and concentration of the extracted RNA was quantified with a NanoDrop 1000 spectrophotometer. Subsequently, 1 µg of total RNA was reverse transcribed using a RevertAid First Strand cDNA Synthesis Kit (Thermo Fisher Scientific, K1622) to obtained cDNAs, which were utilized as the templates for real‐time quantitative PCR (RT‐qPCR) reactions. The qRCR reactions were performed with the FastStart Essential DNA Green Master Mix (Roche) using the Light Cycler 480 Detection System (Roche). All samples were run in triplicate to ensure the accuracy of results. The primers used for qPCR were listed in Table S3 (Supporting Information).

### Immunofluorescence

Under deep anesthesia, mice were perfused with ice‐cold PBS followed by 4% paraformaldehyde (PFA). The brain samples were isolated and fixed in 4% PFA overnight, followed by dehydration in 30% sucrose solution. Then, the brain was cut into 20 µm frozen cryosections. For immunofluorescent staining, the sections were preincubated with PBS containing 0.5% Triton‐X100 and 5% BSA for 20 min and processed with primary antibodies at 4˚C overnight. Subsequently, Alexa Fluor conjugated secondary antibodies were added and incubated for 1 h at room temperature. Primary and secondary antibodies used for immunofluorescence were listed in Table S2 (Supporting Information). Finally, the sections were stained with DAPI for 5 min at room temperature. The penumbra area of brain was imaged using advanced confocal imaging systems including Zeiss 800 Laser Scanning Confocal Microscope, Zeiss 880 Laser Scanning Confocal Microscope with Airyscan, Dragonfly CR‐DFLY‐202 2540 and Nikon A1R N‐SIM N‐STORM Microscope. ImageJ software was used to quantify the fluorescence intensity as well as the co‐localization of tight junction and blood vessels. The level of apoptosis in the ischemic hemisphere was evaluated by terminal deoxynucleotidyl transferase dUTP nick end labeling (TUNEL) using the in situ apoptosis detection kit (C1088, Beyotime) according to the manufacturer's instructions.

### Immunohistochemistry

Adipose tissue was fixed in 4% PFA, embedded with paraffin and then sectioned. Tissue sections were de‐paraffinization and rehydrated before stained with hematoxylin and eosin (H&E). The cross‐section areas of adipose were quantified using the Analyze Particle function in ImageJ.

### Histological Analyses

Quantitative image analysis was performed on maximum projections of 10‐µm‐thick Z‐stack images. For the quantification of extravascular deposition of fibrinogen and albumin, images were converted to binary format, followed by threshold processing and analysis using the Area Integrated Density function in ImageJ. Statistical analysis was conducted on non‐adjacent coronal sections of five mice in each experimental group.

TJ coverage area was determined and normalized by the length of IB4–positive endothelial cells. The quantification of TJ coverage area was presented as the percentage of IB4‐labeled endothelial area associated with ZO‐1/Claudin 5. Four randomly selected fields from five mice per group were analyzed in this study.

### Cytokine Assay

Inflammatory mediators in the ischemic hemisphere were assessed with Proteome Profiler Mouse Cytokine Array Kit (ARY006, R&D system) at 3 days after MCAO according to the manufacturer's instruction. Briefly, the brain was excised and homogenized in PBS containing protease inhibitors. After homogenization, Triton X‐100 was added to reach a final concentration of 1%. The samples were frozen at −70 °C, thawed, and centrifuged at 10,000 g for 5 minutes to remove cellular debris. After quantification of the total protein concentration using Pierce BCA Protein Assay Kit (Thermo Fisher), equal amounts of protein were applied to the test strip. Finally, the greyscale density was determined with Image J (National Institutes of Health), normalized to the reference spots for each sample, and converted to the fold change of the sham group as previous studies described.^[^ ^12^ ^]^

### Cell Culture

bEnd.3 cells were cultured in the Dulbecco's modified Eagle's medium (DMEM) with 10% fetal bovine serum (FBS) at 37 °C and 5% CO_2_ in a humidified incubator. The culture medium was changed every 3 days. To mimic ischemia‐reperfusion injury in vitro, cultured bEnd.3 cells were subjected to transient oxygen glucose deprivation (OGD) for 60 min as previously described.^[^ ^49^ ^]^ Briefly, the cell plates were placed in a hypoxic incubator containing 5% CO_2_ and 95% N_2_ at 37 °C, and the culture medium was replaced with glucose‐free DMEM (Gibco) for 6 hours. Then, the culture medium was switched to DMEM with 10% FBS. BHB (2, 5 or 10 mM), s‐BHB (10 mM), histone deacetylase (HDAC) inhibitor TSA (10 µm, S1045, Selleck), MS‐275 (10 µm, S1053, Selleck) or dimethyl sulfoxide (DMSO) was added to the culture medium 1 hour before OGD and remained in the medium until 24 h after OGD.

### Transcytosis Assay

To test the effect of BHB on bEnd.3 transcytosis, 10‐kDa dextran labeled with Alexa 488 was used to quantify the level of transcytosis. Briefly, the quantification of transcytosis was performed by detecting the fluorescence intensity in endothelial cultural medium on the basolateral side of the transwell after 48 h of coculture using a Tecan microplate reader according to the manufacturer's instructions.

### Chromatin Immunoprecipitation (ChIP)

The binding of H3K9bhb to the promoter of ZO‐1 was examined using a ChIP assay kit (9002, Cell Signaling Technologies). Initially, bEnd.3 cells were treated with 1% formaldehyde on ice to cross‐link the proteins bound to the chromatin DNA, thereby stabilizing the chromatin structure. Following the cross‐linking, the chromatin DNA was sheared into DNA fragments of ≈ 150–500 bp by nuclease. Equal amounts of sheared DNA were utilized for immunoprecipitation with either anti‐H3K9bhb antibodies or an equal amount of IgG. Subsequently, the samples were incubated with protein G magnetic beads, and the immunoprecipitated complex was collected and subjected to reverse cross‐linking. In order to serve as a negative control, an equal amount of sheared DNA without antibody precipitation underwent reverse cross‐linking. The DNA recovered from the reverse cross‐linking was then utilized for qPCR analysis. ChIP–qPCR primers for the ZO‐1 promoter were listed in Table S3 (Supporting Information).

### Statistical Analyses

The investigators who analyzed data was blinded to the group allocation of samples or animals. Age‐ and sex‐matched animals were randomly assigned to experimental groups by generating random numbers through a computer program. All data were displayed as the mean ± S.E.M. from at least three independent experiments. Sample sizes were presented in the figure legends. Statistical analysis between two groups was performed using unpaired t‐test, and one‐ or two‐way analysis of variance (ANOVA) were employed for multiple groups. All data were analyzed using Prism software (GraphPad Software). Statistical significance was taken as a P value less than 0.05, with significance defined as P < 0.05 (*), P < 0.01 (**), and P < 0.001 (***).

## Conflict of Interest

The authors declare no conflict of interest.

## Supporting information

Supporting Information

## Data Availability

The data that support the findings of this study are available on request from the corresponding author. The data are not publicly available due to privacy or ethical restrictions.
