# Supplementary material for: Adaptive Metabolic Responses Facilitate Blood‐Brain Barrier Repair in Ischemic Stroke via BHB‐Mediated Epigenetic Modification of ZO‐1 Expression
Source: Adv Sci (Weinh). 2024 Apr 26;11(25):2400426. doi: 10.1002/advs.202400426 (PMC11220715; doi:10.1002/advs.202400426)
Supplement: Supplementary file 1 — Supporting Information [file ADVS-11-2400426-s001.pdf]

ADVANCED SCIENCE
Open Access

Supporting Information

for *Adv. Sci.*, DOI 10.1002/advs.202400426

Adaptive Metabolic Responses Facilitate Blood-Brain Barrier Repair in Ischemic Stroke via BHB-Mediated Epigenetic Modification of ZO-1 Expression

Ruijie Li, Yilin Liu, Jihao Wu, Xiong Chen, Qiying Lu, Kai Xia, Congyuan Liu, Xin Sui, Yixuan Liu, Yiling Wang, Yuan Qiu, Jinsi Chen, Yi Wang, Ruijun Li, Yucheng Ba, Jiayun Fang, Weijun Huang, Zhengqi Lu, Yanbing Li, Xinxue Liao, Andy Peng Xiang and Yinong Huang**

Adaptive Metabolic Responses Facilitate Blood-Brain Barrier Repair in Ischemic Stroke via BHB-Mediated Epigenetic Modification of ZO-1 Expression.

Supplementary Figures S1-9

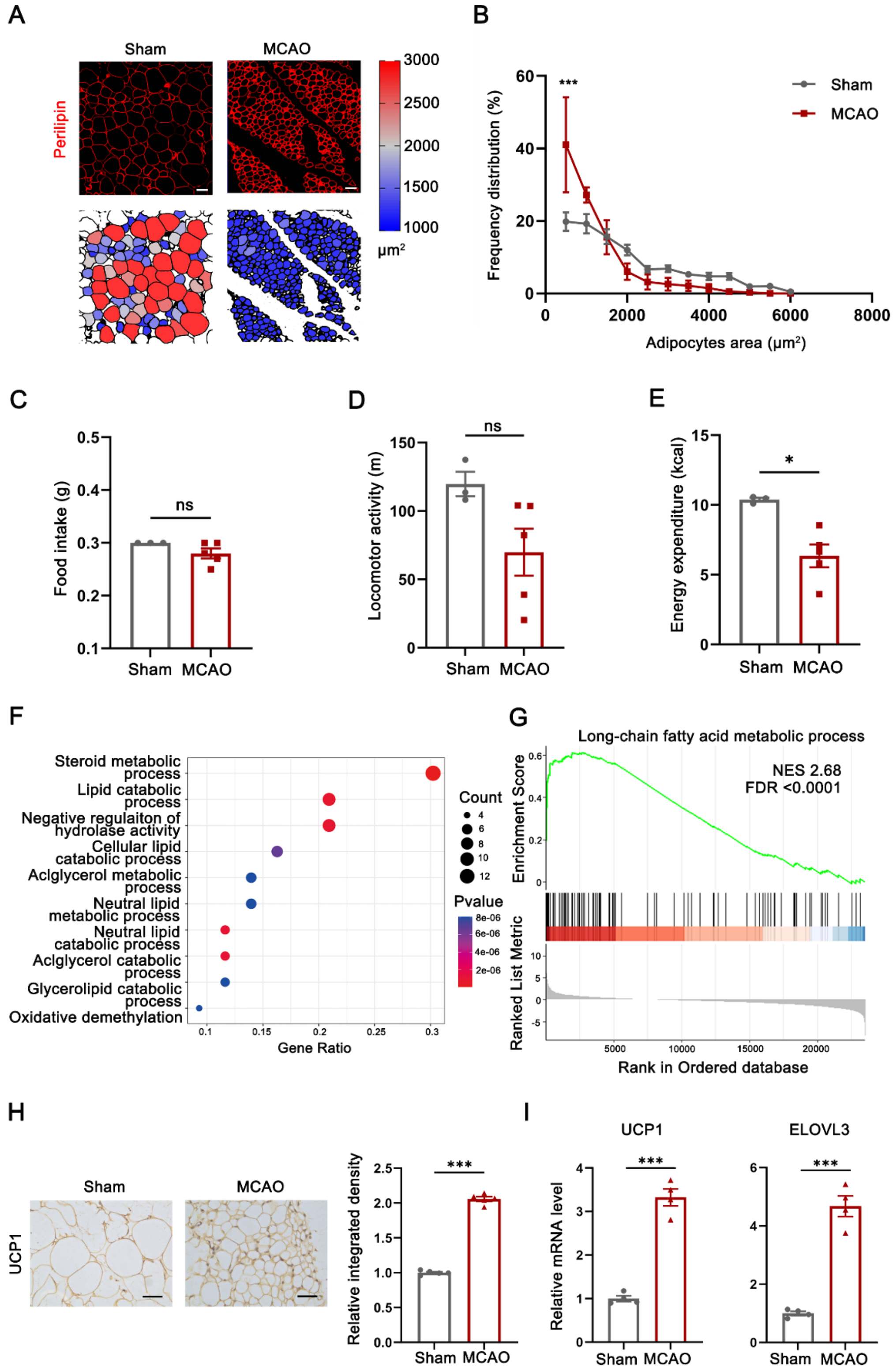

Supplementary Figures S1. Ischemic stroke instigates acute lipolysis and browning in subcutaneous inguinal fat (iWAT).

(A) Immunostaining for perilipin labeling the cross-section area (CSA) of iWAT.

(B) Statistical analysis of adipocyte size frequency distribution. n=4. ***p<0.001, by two-way ANOVA (mean ± S.E.M).

(C-E) Food intake (C), locomotor activity (D) and energy expenditure (E) of indicated groups of mice were determined using indirect calorimetry (IC) 24 hours after surgery. n=3 for sham, n=5 for MCAO. *p<0.05, by unpaired t-test (mean ± S.E.M).

(F) Enrichment analysis of significantly upregulated genes in the iWAT of MCAO mice compared with sham mice.

(G) Gene-Set Enrichment Analysis (GSEA) results highlighted upregulation of long chain fatty acid metabolism in iWAT of MCAO mice compared to sham-operated mice. Abbreviations: NES (normalized enrichment scores), FDR (false discovery rate).

(H) IHC images showing increased UCP1 expression in iWAT after stroke. Scale bar: 25μm. n=5. ***p<0.001, by unpaired t-test (mean ± S.E.M).

(I) mRNA expression levels of browning-related genes were measured in iWAT by qRT-PCR 3 days after stroke. n=4. *p<0.05, **p<0.01, by unpaired t-test (mean ± S.E.M).

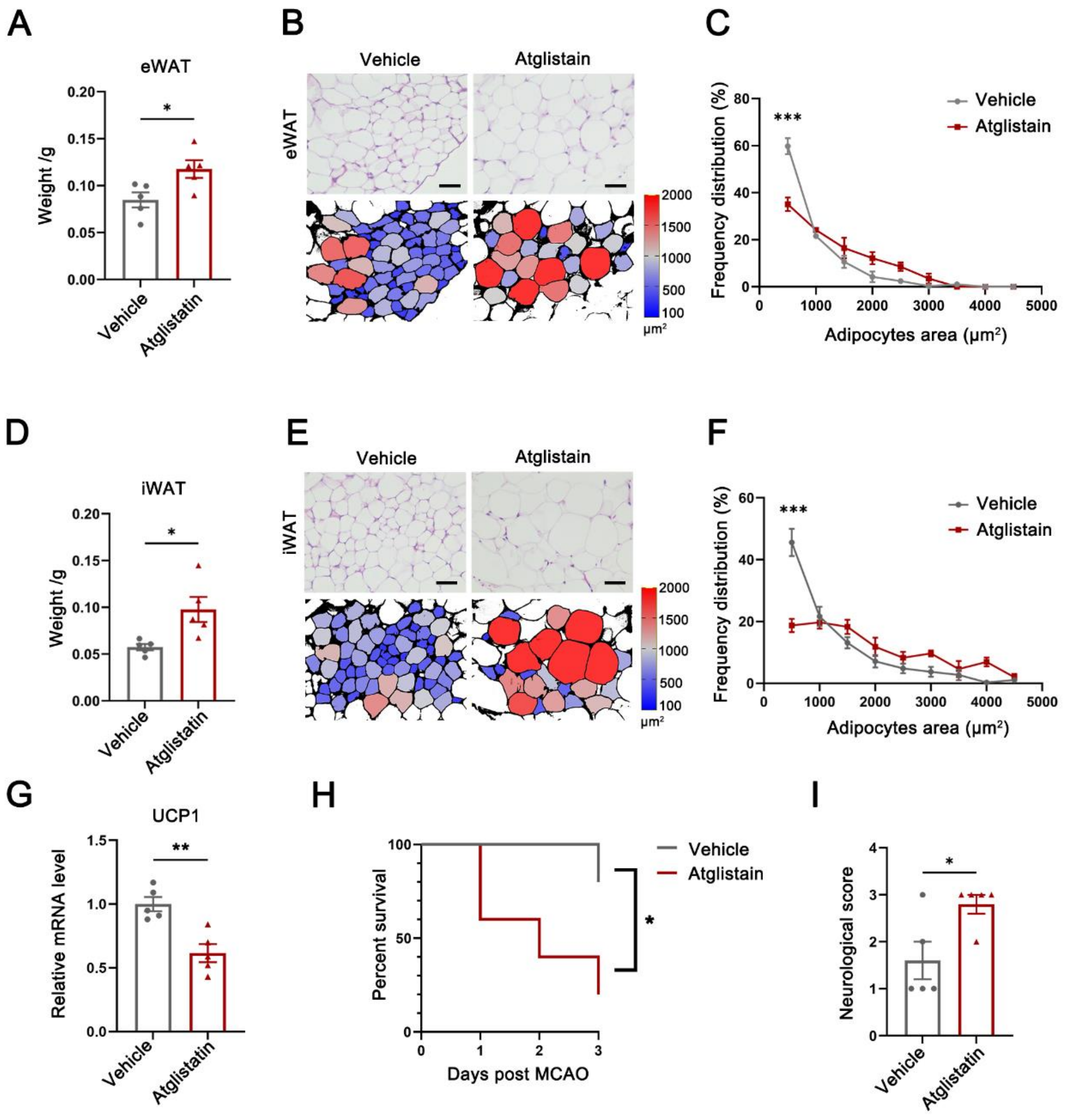

Supplementary Figures S2. Blocking lipolysis aggravates stroke progression.

(A) eWAT weights of vehicle- or atglistatin-treated mice. n=5 *p<0.05, by unpaired t-test (mean ± S.E.M).

(B) Representative images of hematoxylin and eosin (H&E) stained eWAT cross sections. Scale bar: 30μm.

(C) Distribution of adipocyte size in eWAT 3 days after stroke. n=5. ***p<0.001, by two-way ANOVA (mean ± S.E.M).

(D) iWAT weights of vehicle- or atglistatin-treated mice. n=5 *p<0.05, by unpaired t-test (mean ± S.E.M).

(E) Representative images of hematoxylin and eosin (H&E) stained iWAT cross sections. Scale bar: 30μm.

(F) Statistical analysis of adipocyte size frequency distribution of iWAT 3 days after stroke. n=5. ***p<0.001, by two-way ANOVA (mean ± S.E.M).

(G) Relative mRNA expression levels of UCP1 in eWAT of indicated groups. n=5. ***p<0.001, by unpaired t-test (mean ± S.E.M).

(H) Survival curve showing the mortality rate within 3d after stroke in vehicle- and atglistatin-treated mice. n=5. *p<0.05, by log-rank test (mean ± S.E.M).

(I) Neurological deficit scores were evaluated in each group of mice. n=5. *p<0.05, by unpaired t-test (mean ± S.E.M).

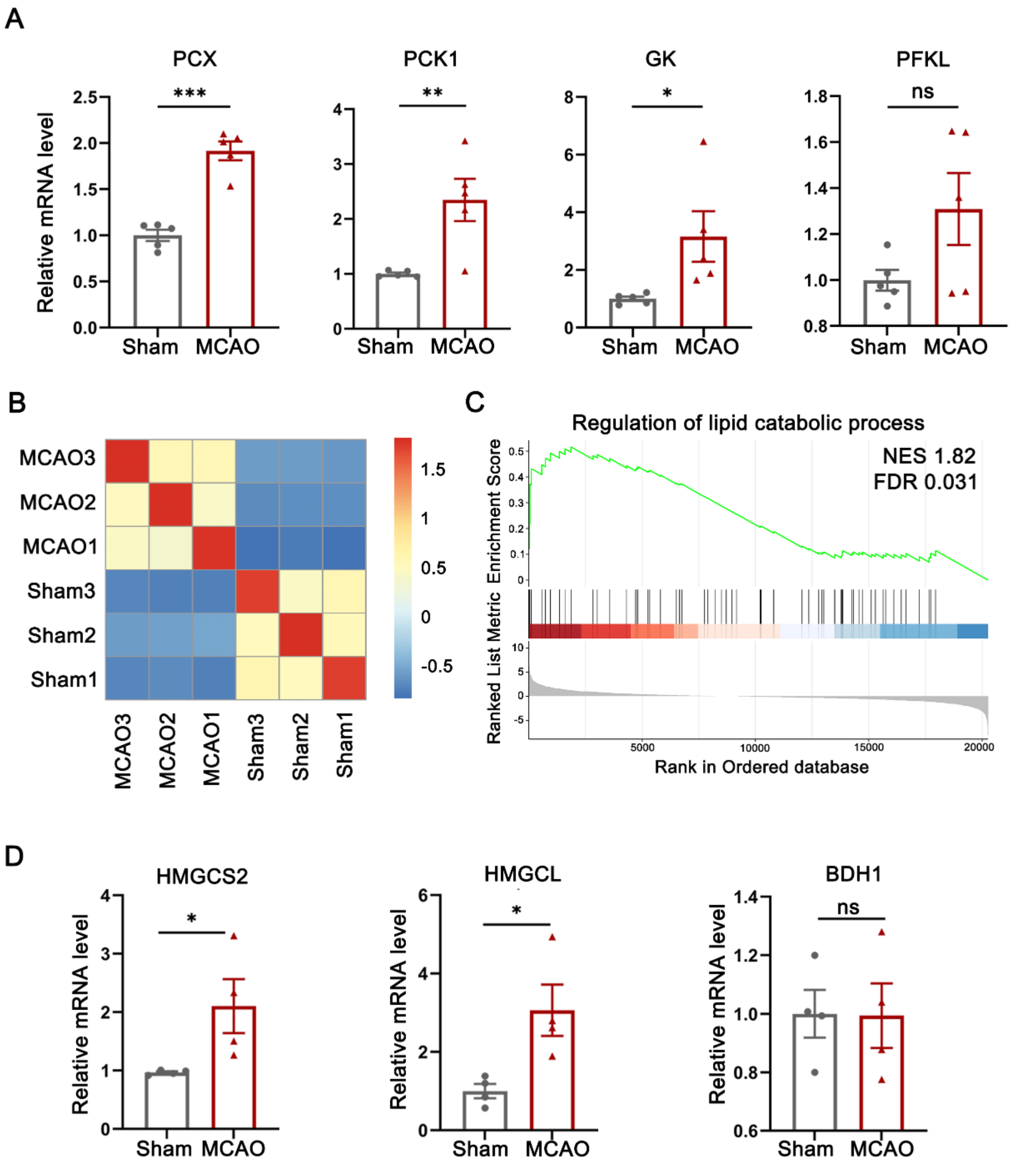

Supplementary Figures S3. Ischemic stroke induces glycogenesis and FA catabolism in the liver.

(A) Hepatic glycogenesis and FA catabolism genes were measured by qRT-PCR 3 days after stroke. n=5. *p<0.05, **p<0.01, ***p<0.001, by unpaired t-test (mean ± S.E.M).

(B) Correlation matrix heatmap showing different transcripts in livers of MCAO mice and sham-operated mice. n=3.

(C) GSEA results highlighted the upregulation of hepatic lipid catabolism in MCAO mice compared with sham-operated mice.

(D) Transcription levels of hepatic ketogenesis-related genes in MCAO mice and sham-operated mice. n=4. *p<0.05, by unpaired t-test (mean ± S.E.M).

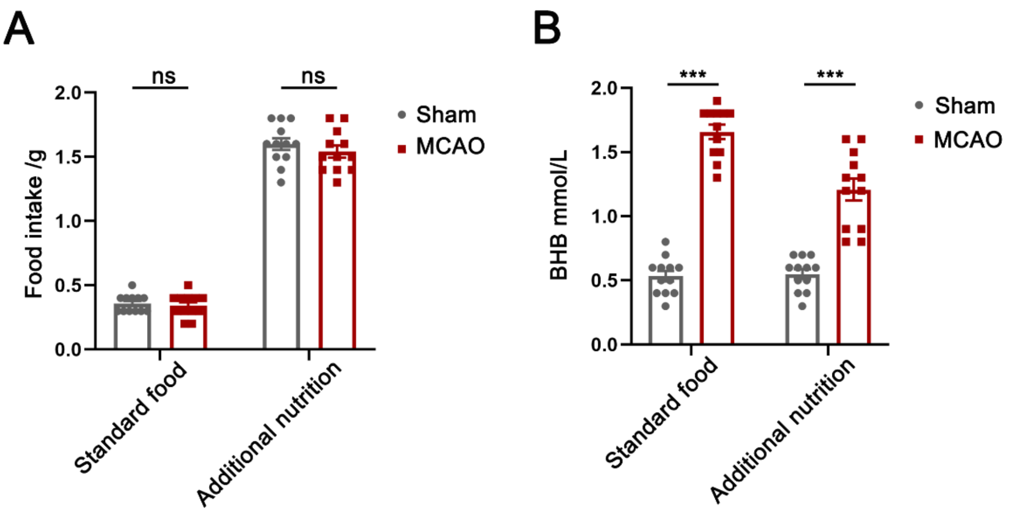

Supplementary Figures S4. Stroke elicits rapid ketogenesis in mice with or without additional nutritional treatment.

(A) The food intake was matched between sham-operated and MCAO mice. Additional jelly-formed food was orally delivered. n=12. n.s. $p > 0.05$, by two-way ANOVA (mean ± S.E.M).

(B) The BHB levels in serum were elevated in MCAO mice with or without additional nutrition support. n=12. ***$p < 0.001$, by two-way ANOVA (mean ± S.E.M).

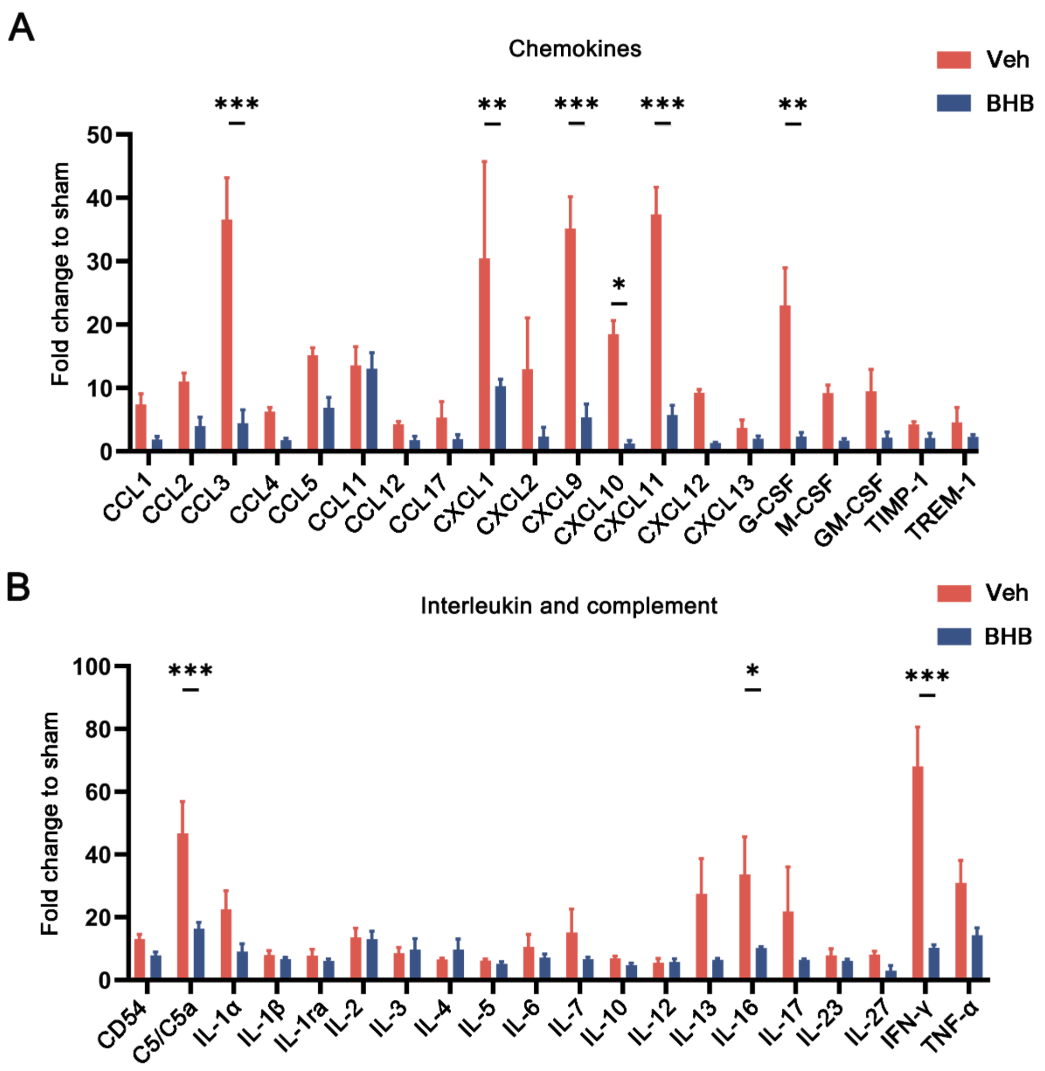

Supplementary Figures S5. BHB treatment reduces inflammatory factors in the ischemic hemisphere.

(A-B) Chemokines, interleukins and complements in ipsilateral hemisphere were evaluated in vehicle- and BHB-treated MCAO mice, respectively. n=3. *$p<0.05$, **$p<0.01$, ***$p<0.001$, by two-way ANOVA (mean ± S.E.M).

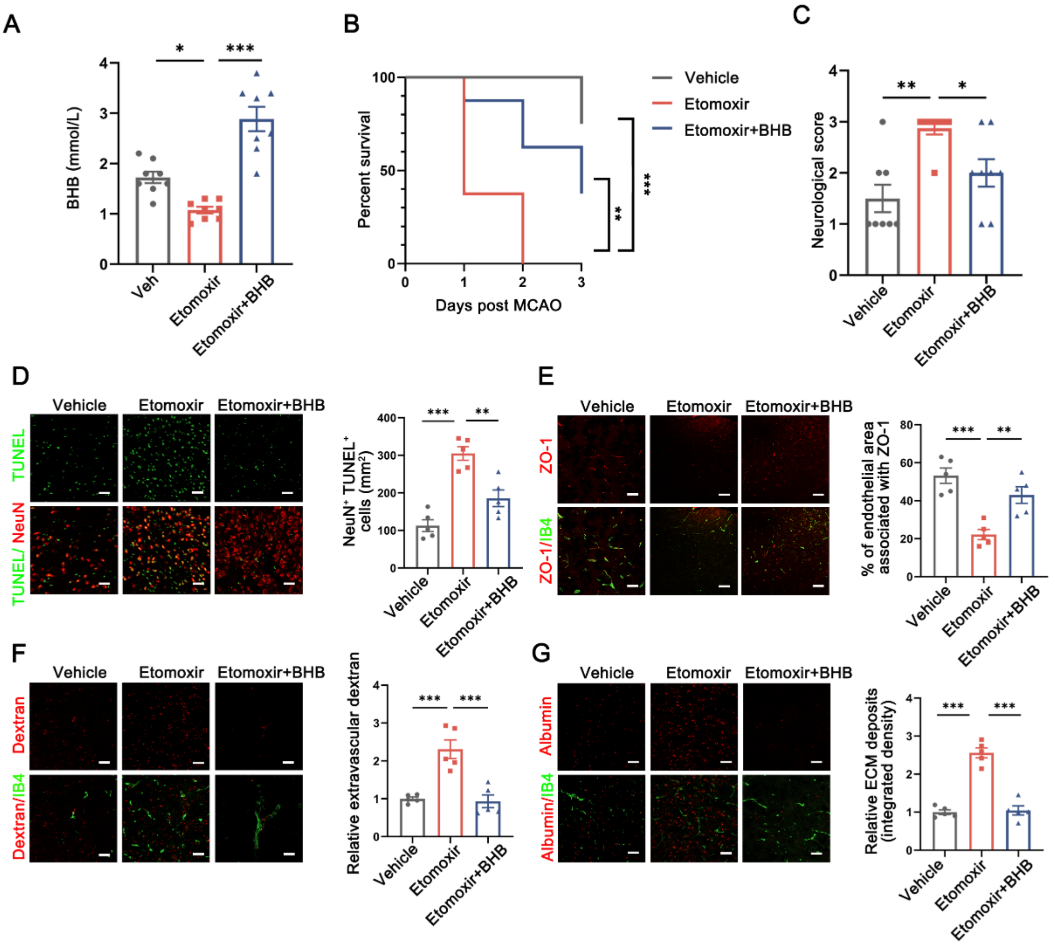

Supplementary Figures S6. BHB supplementation restores BBB leakage and neurological dysfunction aggravated by etomoxir treatment.

(A) Circulating BHB levels were decreased in etomoxir-treated mice. n=8. *$p<0.05$, ***$p<0.001$, by one-way ANOVA (mean ± S.E.M).

(B) BHB treatment partially rescued increased mortality rate induced by etomoxir-mediated FAO disruption. n=8. **$p<0.01$, ***$p<0.001$, by log-rank test (mean ± S.E.M).

(C) Neurological deficit scores were assessed in vehicle- and etomoxir-treated mice with or without BHB supplementation. n=8. *$p<0.05$, **$p<0.01$, by one-way ANOVA (mean ± S.E.M).

(D) Representative confocal images and quantification analysis of $TUNEL^+$ apoptotic neurons in the infarct penumbra of MCAO mice. Scale bar: 50μm. n=5. **$p<0.01$, ***$p<0.001$, by one-way ANOVA (mean ± S.E.M).

(E) Immunofluorescent staining for blood vessels (isolectinB4, IB4) and tight junction

marker (ZO-1) in the peri-infarct region of the ipsilateral hemisphere. Scale bar: 50μm. n=5. **$p<0.01$, ***$p<0.001$, by one-way ANOVA (mean ± S.E.M).

(F-G) Representative confocal images and quantification analysis of dextran (F) and fibrinogen (G) leakage from IB4-labeled vessels. Scale bar: 50μm. n=5. ***$p<0.001$, by one-way ANOVA (mean ± S.E.M).

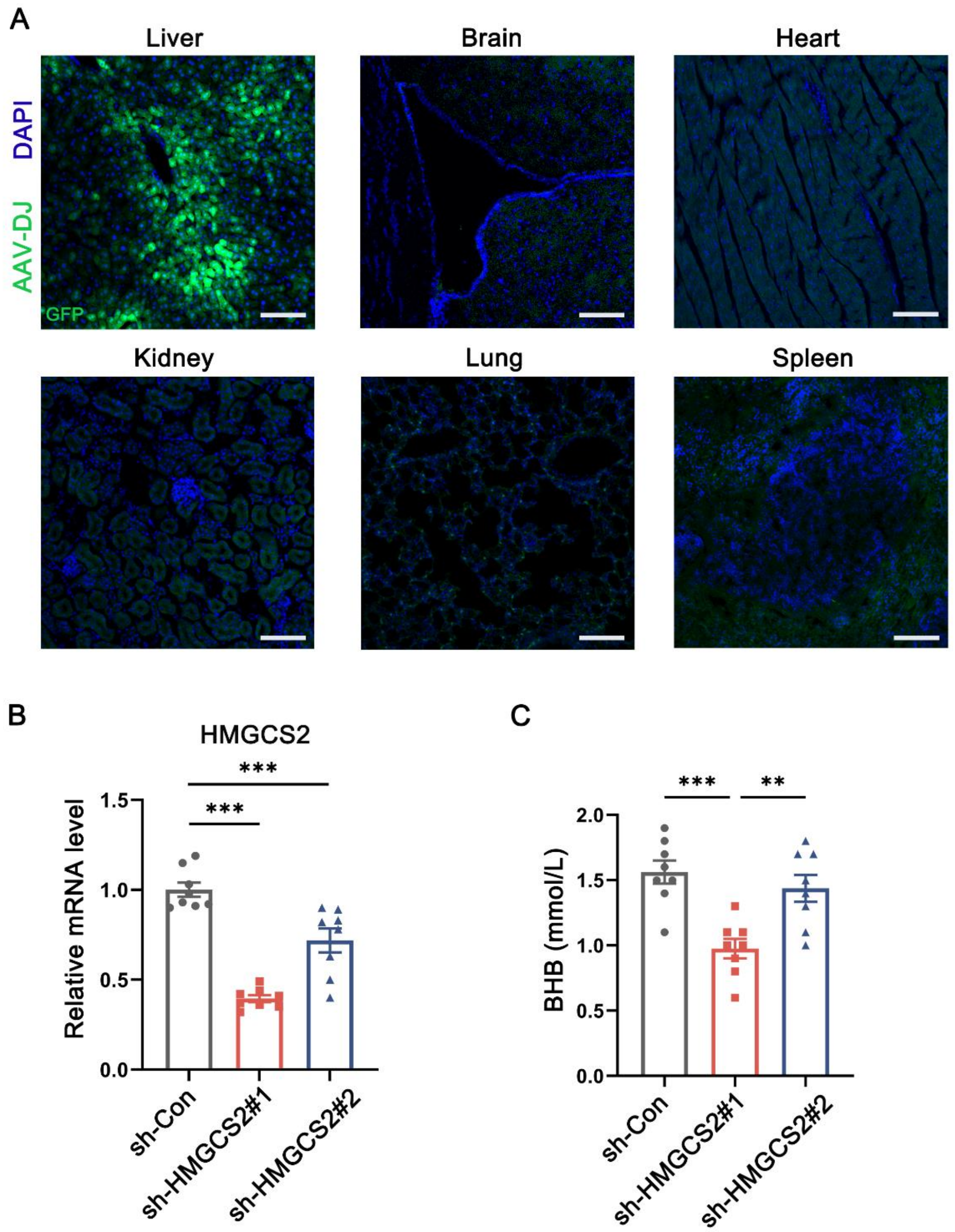

Supplementary Figures S7. The tissue specificity and efficiency of AAV-DJ-mediated HMGCS2 knockdown.

(A) The representative fluorescence images showing focal transduction area in the liver but barely detectable in the brain, heart, kidney, lung, and spleen after systemic injection of AAV-DJ. Scale bar=100μm.

(B) The transcription levels of HMGCS2 in the liver were measured using qRT-PCR, revealing a more pronounced knockdown effect with sh-HMGCS2#1 (referred to as sh-HMGCS2 in the following experiments). n=8. ***p<0.001, by one-way ANOVA (mean ± S.E.M).

(C) Plasma BHB levels of the indicated group. n=8. **p<0.01, ***p<0.001, by one-way ANOVA (mean ± S.E.M).

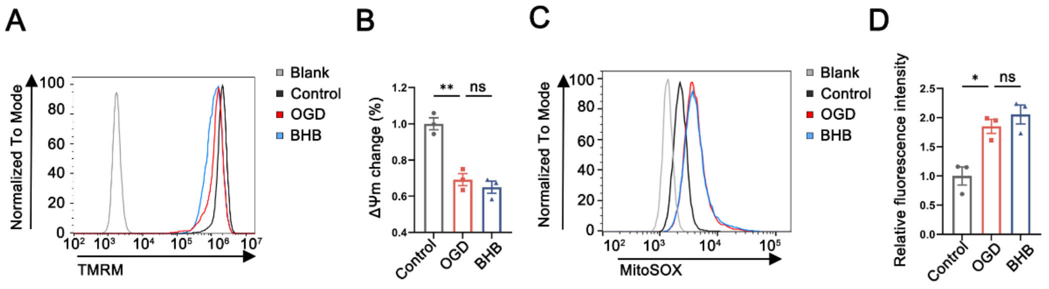

Supplementary Figures S8. BHB treatment fails to alleviate mitochondrial and oxidative stress in endothelial cells.

(A-B) Comparison of MMP using TMRM staining in OGD-treated bEnd.3 cells supplemented with or without BHB. n=3. **p<0.01, by one-way ANOVA (mean ± S.E.M).

(C-D) Density plot analyses (C) and statistical analyses (D) of mitochondrial ROS levels in OGD-treated bEnd.3 cells supplemented with or without BHB. n=3. *p<0.05, by one-way ANOVA (mean ± S.E.M).

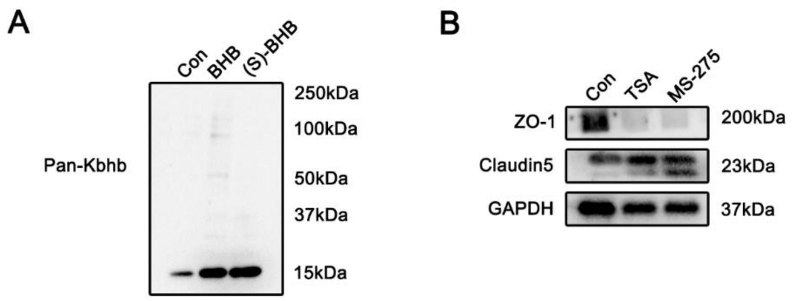

Supplementary Figures S9. BHB regulates ZO-1 expression through β-hydroxybutyrylation rather than acetylation of histone H3.

(A) Western blotting analysis of pan-β-hydroxybutyryllysine (Kbhb) from BHB- or (S)-BHB-treated bEnd.3 cells.

(B) Western blot of ZO-1 and Claudin5 from TSA- or MS-275-treated bEnd.3 cells.
